# Halting Tumor Progression via Novel Non-Hydroxamate Triazole-Based Mannich Bases MMP-2/9 Inhibitors; Design, Microwave-Assisted Synthesis, and Biological Evaluation

**DOI:** 10.3390/ijms221910324

**Published:** 2021-09-25

**Authors:** Fawzia Faleh Albelwi, Mohamed Teleb, Marwa M. Abu-Serie, Mohamed Nabil Abd Al Moaty, Mai S. Alsubaie, Mohamed A. Zakaria, Yeldez El Kilany, Mohamed Reda Aouad, Mohamed Hagar, Nadjet Rezki

**Affiliations:** 1 Department of Chemistry, Faculty of Science, Taibah University, Al-Madinah Al-Munawarah 30002, Saudi Arabia; ffs.chem334@gmail.com (F.F.A.); aouadmohamedreda@yahoo.fr (M.R.A.); 2 Department of Pharmaceutical Chemistry, Faculty of Pharmacy, Alexandria University, Alexandria 21521, Egypt; mohamed.t.ismail@alexu.edu.eg; 3 Medical Biotechnology Department, Genetic Engineering and Biotechnology Research Institute, City of Scientific Research and Technological Applications (SRTA-City), Alexandria 21934, Egypt; marwaelhedaia@gmail.com; 4 Chemistry Department, Faculty of Science, Alexandria University, Alexandria 21321, Egypt; mohamednabil_sc_chem@yahoo.com (M.N.A.A.M.); maisaudalsubaie@gmail.com (M.S.A.); mohamed.zakaria@alexu.edu.eg (M.A.Z.); yeldez244@yahoo.com (Y.E.K.)

**Keywords:** 1,2,3-triazole, 1,2,4-triazole, mannich bases, matrix metalloproteinases-2,9, anticancer

## Abstract

Matrix metalloproteinases (MMPs) are key signaling modulators in the tumor microenvironment. Among MMPs, MMP-2 and MMP-9 are receiving renewed interest as validated druggable targets for halting different tumor progression events. Over the last decades, a diverse range of MMP-2/9 inhibitors has been identified starting from the early hydroxamic acid-based peptidomimetics to the next generation non-hydroxamates. Herein, focused 1,2,4-triazole-1,2,3-triazole molecular hybrids with varying lengths and decorations, mimicking the thematic features of non-hydroxamate inhibitors, were designed and synthesized using efficient protocols and were alkylated with pharmacophoric amines to develop new Mannich bases. After full spectroscopic characterization the newly synthesized triazoles tethering Mannich bases were subjected to safety assessment via MTT assay against normal human fibroblasts, then evaluated for their potential anticancer activities against colon (Caco-2) and breast (MDA-MB 231) cancers. The relatively lengthy bis-Mannich bases **15** and **16** were safer and more potent than 5-fluorouracil with sub-micromolar IC_50_ and promising selectivity to the screened cancer cell lines rather than normal cells. Both compounds upregulated p53 (2–5.6-fold) and suppressed cyclin D expression (0.8–0.2-fold) in the studied cancers, and thus, induced apoptosis. **15** was superior to **16** in terms of cytotoxic activities, p53 induction, and cyclin D suppression. Mechanistically, both were efficient MMP-2/9 inhibitors with comparable potencies to the reference prototype hydroxamate-based MMP inhibitor NNGH at their anticancer IC_50_ concentrations. **15** (IC_50_ = 0.143 µM) was 4-fold more potent than NNGH against MMP-9 with promising selectivity (3.27-fold) over MMP-2, whereas **16** was comparable to NNGH. Concerning MMP-2, **16** (IC_50_ = 0.376 µM) was 1.2-fold more active than **15**. Docking simulations predicted their possible binding modes and highlighted the possible structural determinants of MMP-2/9 inhibitory activities. Computational prediction of their physicochemical properties, ADMET, and drug-likeness metrics revealed acceptable drug-like criteria.

## 1. Introduction 

Medicinal chemistry research is always focusing on modulating the tumor microenvironment, particularly its extracellular matrix, which innately confers cancer growth and metastasis [1,2]. Various crucial cancer progression signaling pathways are dependent on a plethora of proteases released into the tumor extracellular matrix [3]. Matrix metalloproteinases (MMPs) are among the most studied proteases given the fact that most of them have been found dysregulated in nearly all human malignancies [4,5,6]. Twenty-six MMPs have been characterized and correlated as a family of zinc-dependent endopeptidase [7]. The MMPs family was subclassed as collagenases; MMP-1, -8, -13, -18, gelatinases; MMP-2, -9, stromelysins; MMP-3, -10, matrilysins; MMP-7, -26, membrane-type MMPs; MMP-14, -15, -16, -17, -24, -25, and others [8]. All the MMPs family members share nearly the same catalytic domain structure with three a-helixes and five ß-sheets. The domain is characterized by critical active-site zinc coordinated by three histidine residues and five calcium ions. It is divided into C-terminal and N-terminal subdomains by a shallow cleft comprising six binding pockets (S1–S3, and S1′–S3′). S1′ subsite is considered the domain’s selectivity pocket due to its amino acid sequence variations among MMPs [7,9]. A large body of evidence elects MMPs as attractive anticancer targets, being promotors of extracellular matrix turnover, tumor growth, and metastasis [10,11,12]. Their expression levels are tied to the tumor stage and the patient’s prognosis [5]. Among MMPs, gelatinases (MMP-2 and -9) have become the focus of many anticancer research programs being validated as druggable targets for halting cancer progression at different stages [13,14,15,16]. With that, numerous MMPs inhibitors have been introduced over the last decades [14,15,16,17]. Early inhibitors were broad-spectrum mimics of the enzyme’s endogenous ligands capped with the prototypic zinc-binding group hydroxamic acid [18]. Despite their outstanding anticancer potency [18], these peptidomimetics failed in the clinic due to hydroxamic acid-related pharmacokinetics challenges [19] and side effects [13,20,21]. The doubts about hydroxamates suitability directed research strategies to diversify the zinc-binding groups [22]. Although some inhibitors reached clinical trials, they eventually failed to achieve complete success [23]. Consequently, a fundamentally different design approach, utilizing information about the crystal structures of MMPs and computer-aided drug design, adopted introducing non-zinc binding inhibitors for avoiding previous failures. The new molecules can bind to the enzymatic active site while lacking the typical zinc chelation mode [24,25]. Non-zinc binding MMP inhibitors share certain thematic features, such as being long molecules with planar or aromatic linkers. While these architectures tend to be hydrophobic, some hydrophilic interactions are favored by incorporating amino and carbonyl groups [24,26,27]. Accordingly, various lead gelatinases inhibitors were introduced [14,15,16,28]. In the current study, we tailored triazole-based scaffolds mimicking the general theme of MMPs inhibitors while sparing the hydroxamate moiety with its associated drawbacks. It is worth mentioning that over the last few decades, triazoles have received a significant amount of attention [29]. This is because this unique scaffold possesses superior chemical and biological properties. The rational design of such heterocycles in drug discovery purposes has become a prominent area of research, defining medicinal chemistry [30,31]. Hence, we presume to develop new hybrid molecules combining 1,2,4-triazole and 1,2,3-triazoles and incorporating Mannich bases, employing molecular hybridization and click chemistry concepts under both conventional and microwave methods, as part of our ongoing research into the design, synthesis, and biological investigation of such hybrid molecules [32,33,34,35,36,37,38,39,40,41,42,43,44,45,46,47,48]. 

## 2. Design Rationale

**Figure 1:**
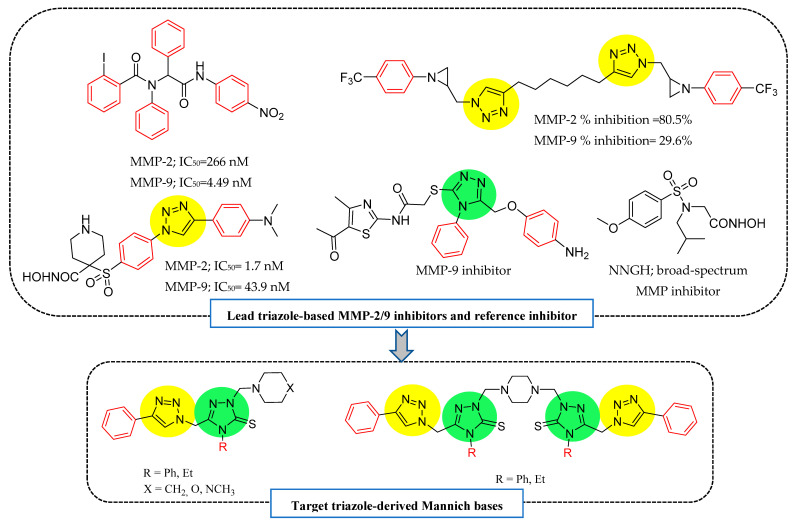
Lead and target MMP-2/9 inhibitors [28,49,50,54].

The design protocol utilized the isomeric triazoles as privileged motifs of many reported lead MMP-2/9 inhibitors [49,50,51,52,53,54,55] (Figure 1). Besides its unique chemical and biological properties, 1,2,3-triazole was rationalized as a non-classical amide isostere [56]. Thus, the designed scaffold comprised three linked aromatic rings with possible hydrophilic interactions. It was also amenable to diversifying its electronic, steric nature via incorporating various aromatic and aliphatic substituents. Importantly, the study was extended to probe the effect of increasing the molecule length on activity through the synthesis of dimeric derivatives (Figure 1). They were preliminarily screened for their potential anticancer activities against breast (MDA-MB 231) and colon (Caco-2) cancers, following an investigation of their safety profiles on normal human lung fibroblasts (Wi-38) via an MTT assay as reported [57,58,59]. The promising derivatives were evaluated for their in vitro MMP-2/9 inhibitory potential. Docking simulations were conducted to predict the structural determinants contributing to receptors interactions. Finally, their physicochemical parameters, ADMET, and drug-likeness profiles were computationally predicted.

## 3. Results and Discussion 

### 3.1. Chemistry 

**Scheme 1:**
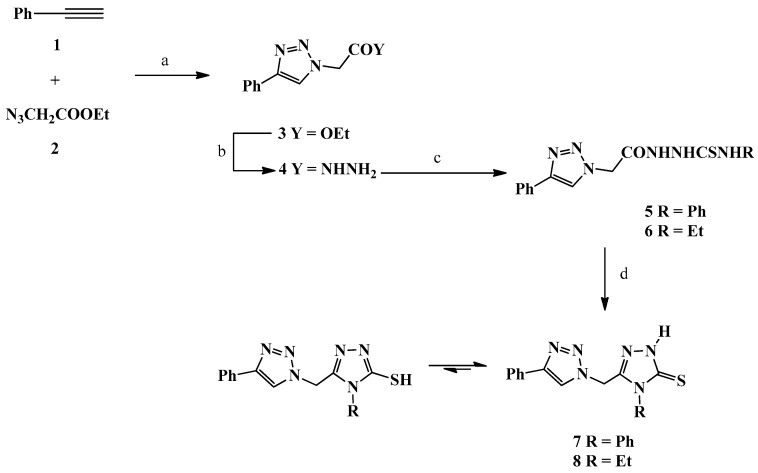
Synthesis 4-phenyl-1,2,3-triazole and 4-substituted 1,2,4-triazole-5-thione hybrids **7** and **8**. Reagents and conditions: (**a**) CuSO_4_, Na L-ascorbate, *t*-BuOH, H_2_O, MW 3 min; (**b**) NH_2_NH_2_.H_2_O, MW 2 min; (**c**) RNCS, EtOH, MW 3 min; (**d**) 10% NaOH, MW 4 min.

**Scheme 2:**
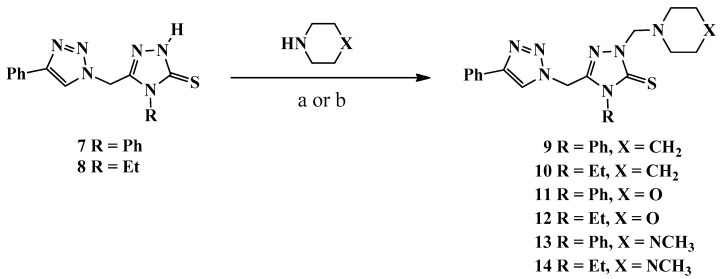
Synthesis of the target Mannich bases **9**–**14**. Reagents and conditions: (**a**) HCHO, secondary amine, EtOH, reflux 16–20 h; (**b**) HCHO, secondary amine, EtOH, MW 5–6 min.

**Scheme 3:**
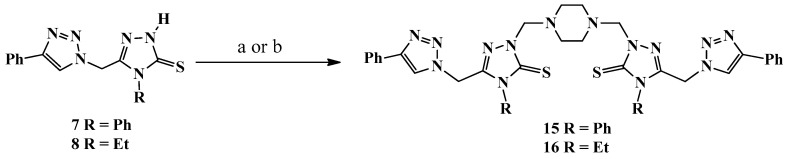
Synthesis of the bis Mannich bases **15** and **16**. Reagents and conditions: (**a**) HCHO, piperazine, EtOH, reflux 20 h; (**b**) HCHO, piperazine, EtOH, MW 6 min.

The synthetic approach adopted for the synthesis of the targeted 1,2,3-triazole-1,2,4- triazole molecular hybrids is depicted in Scheme 1, Scheme 2 and Scheme 3. The Cu(I)-catalyzed 1,3-Dipolar cycloaddition reaction (CuAAC) is a promising methodology for the synthesis of 1,4-disubstituted-1,2,3-triazole scaffold with high regioselectivity and efficiency. Thus, 1,3-dipolar cycloaddition of phenyl acetylene **1** with ethyl azido acetate **2**, in the presence of CuSO_4_·5H_2_O and sodium L-ascorbate, resulted in the formation of 4-phenyl-1,2,3-triazole derivative **3** carrying ester functionality in 91% yield. When the click reaction was carried out under microwave irradiation, the click adduct **3** was obtained in 3 min and 98% yield. Hydrazinolysis of ester **3** with hydrazine hydrate under MW irradiation for 2 min gave the acid hydrazide **4** in excellent yield (97%). The thermal hydrazinolysis required heating under reflux for 4 h to afford the same acid hydrazide **4** in 89% yield. The acid thiosemicarbazides **5** and **6** were obtained in 95–96% yield through the microwave-assisted condensation of the synthesized acid hydrazide **4** with phenyl and/or ethyl isothiocyanates for 3 min. Under conventional heating for 4 h, compounds **5** and **6** were obtained in 88 and 86%, respectively. Furthermore, intramolecular dehydrative cyclization of the resulted acid thiosemicarbazides **5** and **6** was carried out in basic media (10% NaOH), under reflux for four hours, to get the desired 1,2,4-triazoles **7** and **8** in good yields (88–86%). Microwave-assisted intramolecular cyclization required only 4 min to afford the targeted 1,2,3-triazole-1,2,4-triazole molecular hybrids **7** and **8** in excellent yields (96–97%) (Table 1).

The structures of **7** and **8** were elucidated from their NMR spectra, where the ^1^H-NMR spectra showed the presence of a distinct singlet at δ_H_ 14.11–13.93 ppm attributed to the 1,2,4-triazolyl-NH proton. The ^13^C-NMR spectra showed the presence of a characteristic downfield signal at δ_C_ 169.15–167.60 ppm corresponding to the C=S group; these data confirmed the presence of the 1,2,4-triazoles **7** and **8** in their thione form rather than the thiol analogs. Moreover, the methylene protons between the two triazole rings were resonated as a singlet peak at δ_H_ 5.93–5.66 ppm, which are correlated to its carbon at δ_C_ 44.99–44.55 ppm in the ^13^C-NMR spectra. Additionally, H-5 of the 1,2,3-triazolyl ring was observed as a singlet peak at δ_H_ 8.71–8.25 ppm correlated to C-5 at 122.68–122.55 ppm. 

Our next approach was the Mannich reaction of the synthesized compounds **7** and **8** to afford the targeted Mannich bases encompassing triazoles molecular conjugates **9**–**14**. Thus, aminomethylation reaction of 4-substituted-3-((4-phenyl-1*H*-1,2,3-triazol-1-yl)methyl)-1*H*-1,2,4-triazole-5(4*H*)-thione **7** and/or **8** with formaldehyde and the appropriate secondary amine (piperazine, morpholine, and *N*-methyl piperazine), in ethanol under reflux for 16–20 h, afforded the N-Mannich bases **9**–**14** in 83–86% yields (Scheme 2). The formation of the N-aminomethylated base was due to the formation of the immonium salt that subsequently attacks the N-1 of 1,2,4-triazole ring to give the regioselective N-Mannich bases **9**–**14**. The yields of the target hybrids were improved when the reaction proceeded under microwave irradiation, where the time was significantly reduced to 5–6 min and the yield was increased to 92–96% (Table 1).

The structure elucidation of the synthesized Mannich bases **9**–**14** was established based on their ^1^H- and ^13^C-NMR spectra. All their ^13^C-NMR spectra revealed the presence of a characteristic signal corresponding to the C=S at δ_C_ 170.10–168.37 ppm that distinguished the thione form of the synthesized compounds. 

Moreover, the investigation of their ^1^H-NMR spectra supported the formation of such bases by the disappearance of the 1,2,4-triazolyl-NH protons of **9**–**14** compared to their starting material **7** and **8**, and the appearance of new singlet around δ_H_ 5.11–4.98 ppm belonging to the N-**CH**_2_-N, which were resonated at δ_C_ 70.40–68.90 ppm in their ^13^C-NMR spectra, confirming the success of the aminomethylation reaction. The remaining protons and carbons were recorded in their expected area; the methylene (-**CH**_2_) linking the two triazolic rings were resonated as a singlet at δ_H_ 6.00–5.71 ppm, correlated with the signal observed at δ_C_ 44.82–44.35. In addition, H-5 of the 1,2,3-triazolyl proton was observed as a singlet between at δ_H_ 8.76–8.21 ppm in a correlation to a signal at δ_C_ 122.75–122.50 ppm in their ^13^C-NMR spectra, respectively. Other protons and carbons corresponding to the phenyl ring or ethyl group in addition to the secondary amine (piperidine, morpholine, and *N*-methyl piperazine) moiety were investigated in the experimental section. 

Under the same optimized conditions, the reaction of two equivalents of triazole **7** and/or **8** with one equivalent of piperazine and formaldehyde in refluxing ethanol for 20 h afforded the respective bis-aminomethylated Mannich bases **15** and **16** in 81–82% yield (Scheme 3). Under microwave irradiation, only 6 min were required to yield the same Mannich bases **15** and **16** in 92% yield. The NMR spectra of the bis aminomethylated Mannich base **15** showed the presence of an extra signal for some protons and carbons, which may be due to the unsymmetrical conformation of the structure with regard to its minimized energy. Thus, the investigation of its spectral data revealed the presence of two distinguishable singlets resonating at δ_H_ 5.71 and 5.68 ppm assigned to the two **CH**_2_ linker groups between the 1,2,3- and 1,2,4-triazole moieties and correlated with the signal resonated at δ_C_ 42.91 ppm in the ^13^C-NMR spectrum. In addition, a characteristic singlet was observed at δ_H_ 4.92 ppm, integrating with four protons assigned to the two N-**CH**_2_-N groups and correlated with the signal appeared at δ_C_ 53.59 ppm. Moreover, two singlets were recorded at δ_H_ 8.46 and 8.28 ppm attributed to the **H**-5 protons of the two 1,2,3-triazole rings and associated to signals at δ_C_ 123.01 and 122.61 ppm. The presence of two signals at δ_C_ 169.67 and 169.11 ppm proved the presence of two thione carbons (C=S) supporting the *N*-aminomethylation rather than *S*-aminomethylation. On the other hand, the NMR spectra of compound **16** showed equivalent signals, confirming its symmetric structure, where a singlet peak integrating two protons was observed at δ_H_ 8.73 ppm, corresponding to H-5 of the two 1,2,3-triazole moieties and correlated to the signal resonating at δ_C_ 122.71 ppm. The linker methylene protons (**CH**_2_) which combine the 1,2,3-triazole and the 1,2,4-triazole rings were resonated at δ_H_ 5.97 ppm as a singlet peak integrating with four protons and correlated with signal resonated at δ_H_ 44.35 ppm in its ^13^C-NMR spectrum. The N-**CH**_2_-N protons were observed at δ_H_ 4.98 ppm as a singlet and correlated with that appeared at δ_C_ 68.93 ppm. The signal recorded at δ_C_ 168.40 ppm was attributed to two equivalent thione (C=S) carbons, confirming the symmetrical structure of **16**. The remaining protons and carbons were investigated from the NMR spectra and represented in the experimental section. It should be noted that the unsymmetric conformation of the bis-Mannich base **15** may be due to the presence of the *N*-phenyl groups that affect the stability of the structure and, consequently, its conformation due to their steric hindrance rather than the ethyl groups in compound **16**. 

### 3.2. Biology 

#### 3.2.1. Cytotoxicity Screening 

All the newly synthesized derivatives were firstly screened for cytotoxic activities on normal lung fibroblasts (Wi-38) for evaluating their safety profiles. Then, they were subjected to anticancer revaluation against two selected human tumors (MDA-MB 231 and Caco-2) compared 5-fluorouracil as reference chemotherapy via MTT assay (Table 2). All the studied compounds were more active than the reference against MDA-MB 231, with **15** and **16** at the top of the list recording submicromolar IC_50_, followed by **13**, **12**, **10**, **9**, **14**, and **11**, respectively. On the other hand, only **15** (IC_50_; 0.26 µM) and **16** (IC_50_; 0.39 µM) were superior to 5-fluorouracil against Caco-2 cells. Moreover, **13** (IC_50;_ 0.61 µM) was nearly comparable to the reference. The remainder compounds were relatively moderate with IC_50_, ranging from 1.63–3.31 µM. The assessment of the compounds’ selectivity towards tumor cells rather than the normal ones is key to real evaluation. Herein, the selectivity index (SI) was calculated for each compound as the ratio of its IC_50_ against normal and cancer cells (Table 2). It is generally accepted that SI ≥3 acknowledges considerable selectivity of the compound to cancer cells [64]. Interestingly, the most active derivative among the group **15** recorded the highest SI were against Caco-2 (SI; 4.13) and MDA-MB 231 (SI; 3.56) cells, exceeding the acceptable SI limit against both cancer cell lines. Accordingly, the two compounds 15 and 16 were selected for further mechanistic studies, whereas other derivatives were considered beyond the acceptable selectivity.

**Figure 2:**
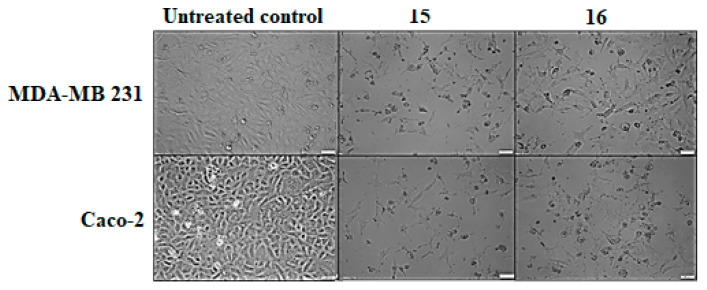
Morphological changes of MDA-MB 231 and Caco-2 cells after 72 h treatment with 15 and 16 at 3 µM.

MDA-MB 231 and Caco-2 cells were examined after 72 h treatment with **15** and **16** at their EC_100_ concentrations (Table 2) in comparison with the control untreated cancer cells (Figure 2). The treated cancer cells lost their characteristic shape. Severe shrinkage was observed reflecting the potent anticancer potential of the studied compounds [65].

#### 3.2.2. Apoptosis Induction Evaluation

The apoptotic induction efficacy of **15** and **16** was evaluated by quantifying the elevation in the tumor suppressor gene p53 expression and suppression in oncogene (cyclin D)-mediated cell cycle progression via quantitative real-time PCR analysis. 

##### Quantitative Real-Time PCR Analysis of p53 Gene 

The tumor suppressor p53 gene is one of the main apoptosis induction markers. It is mutated in 50% of human cancers, including breast and colon. Its upregulation leads to induction of cell cycle arrest and apoptosis [66,67]. Although MDA MB-231 cells carry a mutated p53 gene (R280K mutation), mechanistic studies demonstrated that treatment with anticancer agents inducing p53-dependent intrinsic apoptosis (6-gingerol) resulted in p53 expression and quick apoptosis execution, supporting the hypothesis that p53 in MDA MB-231 is indeed a gain-of-function mutant [68,69,70]. Similarly, the expression of apoptosis regulatory genes evaluated by PCR following treatment of Caco-2 cells with some apoptotic inducers (*Drimia calcarata* bulb extracts) revealed p53 upregulation, even though p53 was not detected in the untreated cells [71].

**Figure 3:**
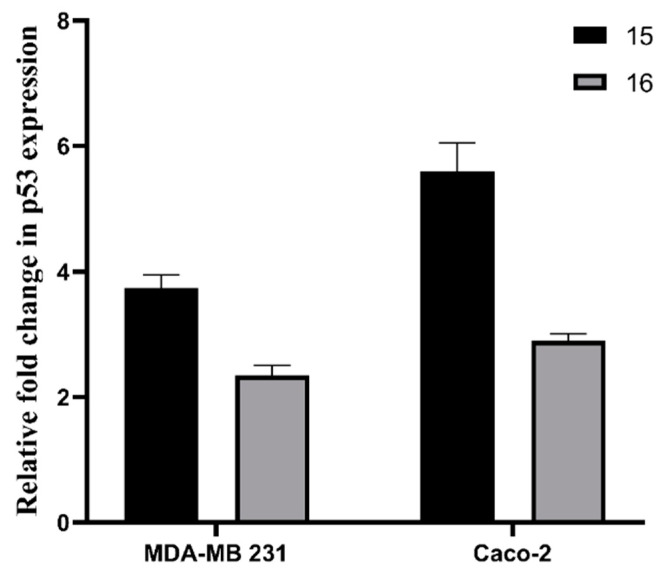
Relative fold change in p53 expression in MDA-MB 231 and Caco-2 cells after treatment with **15** and **16** at their respective IC_50_ concentrations. All values are expressed as mean ± SEM.

In the current study, Figure 3 demonstrates that **15** and **16** upregulated p53 in the studied cancer cell lines by more than 2-fold. Moreover, **15** exhibited higher potential for enhancing p53 expression (3.74 ± 0.21 and 5.59 ± 0.46-fold) than **16** (2.35 ± 0.16 and 2.89 ± 0.11-fold) in MDA-MB 231 and Caco-2 cells, respectively. 

##### Quantitative Real-Time PCR Analysis of Cyclin D

Cyclin D is the key mediator for transition from the G1 phase of the cell cycle to the S phase via activation of cyclin-dependent kinase. Accordingly, halting its expression leads to cell cycle arrest and apoptosis induction [72]. 

**Figure 4:**
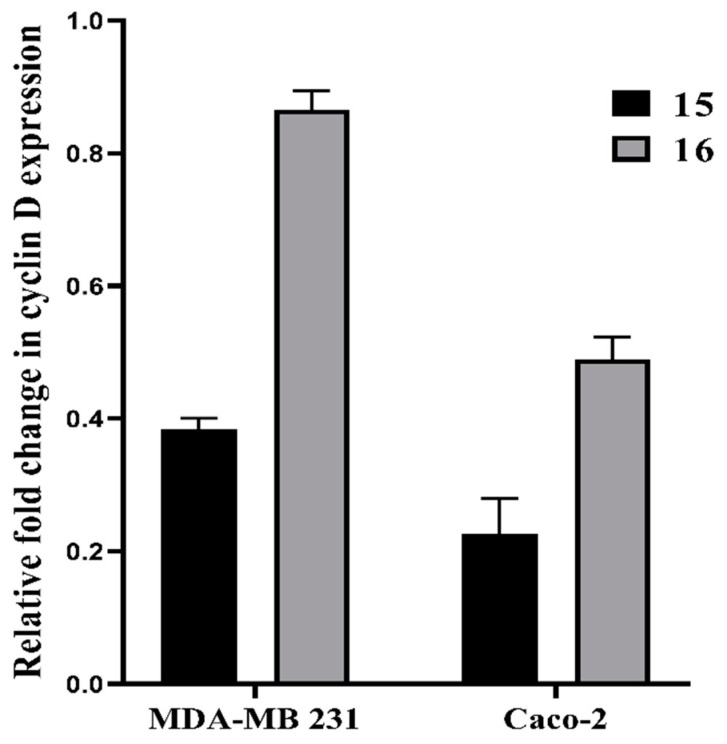
Relative fold change in cyclin D expression in MDA-MB 231 and Caco-2 cells after treatment with **15** and **16** at their respective IC_50_ concentrations. All values are expressed as mean ± SEM.

Quantitative real-time PCR analysis revealed that both **15** and **16** suppressed cyclin D (Figure 4). The detected relative fold decrease of cyclin D expression levels in **15**-treated MDA-MB 231 and Caco-2 cells (0.23 ± 0.05 and 0.38 ± 0.02, respectively) was lower than that in the **16**-treated cancer cells (0.49 ± 0.034 and 0.87 ± 0.03, respectively). 

#### 3.2.3. MMP-2/9 Inhibition

The inhibitory potential of the hit anticancer derivatives **15** and **16** against MMP-2 and -9 was evaluated in terms of the percentage of inhibition at 0.4 µM (≈anticancer IC_50_; Table 2) in comparison to the reference prototypic inhibitor *N*-Isobutyl-*N*-(4-methoxyphenylsulfonyl)glycyl hydroxamic acid (NNGH) (Figure 1) through a colorimetric assay in a 96-well microplate format using a chromogenic substrate (Ac-PLG-[2-mercapto-4-methyl-pentanoyl]-LG-OC_2_H_5_) (Table 3). An MMP-2 or MMP-9 cleavage site bond is replaced by a thioester in the included thiopeptide, which affords sulfhydryl group upon hydrolysis. The sulfhydryl moiety reacts with Ellman’s reagent to produce 2-nitro-5-thiobenzoic acid, which is detectable at 412 nm according to the kits manufacturers’ protocol. Then, the same above-mentioned procedure was performed at serial concentrations of **15**, **16,** and the reference inhibitor NNGH to calculate their IC_50_ values against MMP-2 and -9 (Table 3). 

Interestingly, **15** recorded higher MMP-9% inhibition than both **16** and the reference inhibitor NNGH at its anticancer IC_50_ concentration. At similar concentrations, **16** was comparable to NNGH. As for MMP-2, an opposite inhibition pattern was detected, where **16** was superior to **15**, and both were less active than NNGH. IC_50_ determination results provided more clarity about the inhibition profiles, where **15** was approximately 4-fold more active than **16** and NNGH against MMP-9, while **16** was comparable to NNGH. On the other hand, **16** was slightly (1.2-fold) more active than **15** against MMP-2 inhibitor. In light of the mentioned results, **15** was 3.27-fold more active against MMP-9 than MMP-2, highlighting its promising selectivity towards MMP-9 compared to **16** and NNGH. 

### 3.3. Molecular Modeling

#### 3.3.1. Docking Simulations 

Docking was performed by MOE 2015.10 [73] on the promising compounds **15** and **16** to predict their major determinants towards MMP-2/9 binding sites and the structural features crucial for activity and selectivity. The crystal structures of the two gelatinases, MMP-2 (PDB code: 1HOV [74]) and MMP-9 (PDB code: 1GKC [75]), were retrieved from the protein data bank to derive the receptor models. Unnecessary residues and solvent molecules were eliminated, then the MOE “QuickPrep” module was utilized for structure preparation with the default settings. The studied compounds **15** and **16** were built in silico and energy minimized according to the default geometry optimization settings. Taking into account that the MMP active site may be biased to the hydroxamate-based co-crystallized ligand, the possible binding sites encompassing the key residues were located using the MOE ‘Site Finder’ feature. Docking of the studied compounds was firstly run with various fitting protocols to record the best docking scores and binding interactions. The best binding modes were computed by rigid docking employing the Triangle Matcher placement method and London dG scoring function. The selected protocol was validated by re-docking the co-crystallized ligand in the active site and reproducing most of the key experimental interactions at acceptable RMSD.

**Figure 5:**
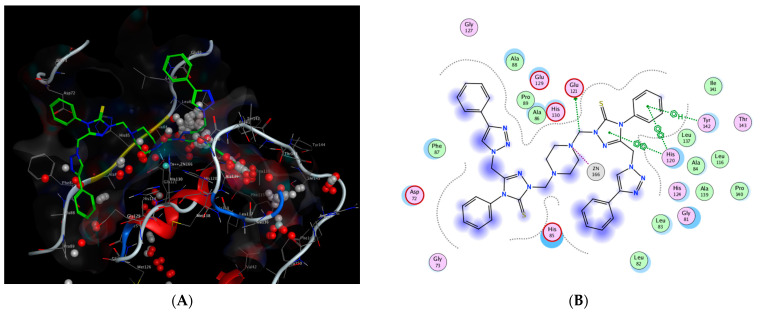

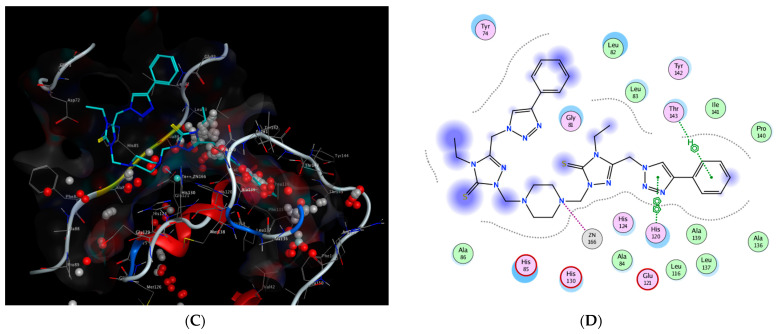
(**A**) Three-dimensional binding mode of **15** (green sticks), (**B**) 2D binding mode of **15**, (**C**) 3D binding mode of **16** (cyan sticks), and (**D**) 2D binding mode of **16** in MMP-2 catalytic domain (PDB ID: 1HOV [74]).

In MMP-2 docking (Figure 5), the computed scores (binding energies) of the top docking poses corresponding to the lowest energy conformers of **15** and **16** were approximately similar (ΔG = −12.133 and −11.900 Kcal/moL, respectively). Both compounds resided well in the active site towards the S1′ pocket forming π-π stacking between His120 and the triazole rings, H-π interactions between Tyr 142 and the phenyl rings substituents of the triazoles, as well as interactions between the active site zinc ion and the piperazinyl nitrogens. In **15,** additional π-π stacking between His120 and the phenyl ring on the 1,2,4-triazole ring and H-bonding between Glu121 and the triazole-piperazine linker methylene were detected.

**Figure 6:**
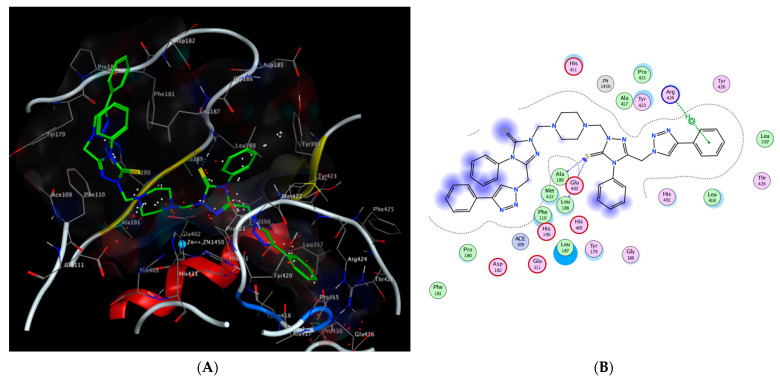

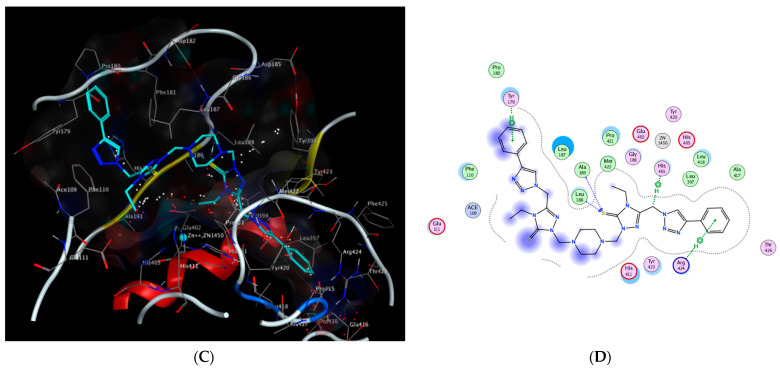
(**A**) Three-dimensional binding mode of **15** (green sticks), (**B**) 2D binding mode of **15**, (**C**) 3D binding mode of **16** (cyan sticks), and (**D**) 2D binding mode of **16** in MMP-9 catalytic (PDB code: 1GKC [75]).

As for MMP-9 (Figure 6), the computed scores of the top docking poses for **15** and **16** well resembled the experimental outcome so far, (ΔG = −10.102 and −9.522 Kcal/moL, respectively). Both compounds fitted into the MMP-9 catalytic domain and looped into the proximity of the S1′ pocket. They exhibited hydrogen bonding interactions between the 1,2,4-triazole thione, Leu188, and Ala189. H-π interactions were also detected between the 1,2,3-triazole phenyl and Arg424. Additional H-π interactions were predicted between the methylene, linking the isomeric triazoles of **16** and the active site His 401.

#### 3.3.2. ADMET and Drug-Likeness Prediction 

In silico computation of ADMET and drug-likeness parameters has attracted considerable interest in the recent medicinal chemistry research programs as a useful tool for identifying lead compounds. Herein, various physicochemical properties formulating the drug-likeness parameters were predicted for our hit compounds **15** and **16** utilizing *SwissADME* [76], *Pre-ADMET* [77], and PROTOX [78] servers (Table 4). Both compounds showed acceptable predicted drug-like bioavailability according to the parameters used by Lipinski [79]. Two violations were detected according to the parameters used by Veber [80] and only one according to the parameters used by Egan [81]. Both compounds were predicted to possess high intestinal absorption (>98%), moderate CNS absorption, medium Caco2 model permeability, low MDCK model ones, high plasma proteins binding, and weak aqueous solubility. They lack cytochromes P450 2D6 (CYP2D6) inhibition activities but not CYP3A4 according to *Pre-ADMET* [77]. Moreover, the toxicity predictor software PROTOX [78] computed their acute oral toxicity in rodents in terms of their average lethal dose (LD_50_) as 1000 mg/Kg; hence, both compounds were classified according to the Globally Harmonized System of Classification and Labeling of Chemicals (GHS) as class IV. Collectively, both **15** and **16** could be viewed as promising drug-like molecules. 

### 3.4. Structure–Activity Relationship

The cytotoxicity pattern of the screened Mannich bases **9**–**16**, reported in Table 2, suggests a key role of the molecule length and hydrophobicity in determining potency and selectivity. This pool of compounds showed promising anticancer activities. Notably, the bis Mannich bases **15** and **16** displayed the highest activity and selectivity compared to their monomeric precursors as well as the reference chemotherapy. The phenyl substituted bis Mannich base **15** was superior to the ethyl substituted derivative **16**. In most cases, a similar observation was detected when comparing the cytotoxic activities of the phenyl substituted Mannich bases **9**, **11,** and **13**, with the ethyl substituted analogs **10**, **12,** and **14**, highlighting the preference of the electronic, hydrophobic, and steric effects of aromatic substituents to anticancer activity in these particular positions. Another driver of the anticancer potency was the heterocyclic amine at the 1,2,4-triazole motif, where *N*-methyl piperazine conferred, in most derivatives, superior activities especially against Caco-2 cells compared to morpholine and piperidine. Docking simulations of the most promising derivatives **15** and **16** into the MMP-2 and -9 active sites demonstrated that both compounds resided well, and highlighted the significance of the compound’s length on activity (Table 3). The 4-phenyl substituents on the 1,2,4-triazole motifs conferred higher MMP-9 selectivity to the bis Mannich base scaffold compared to the ethyl substituents. This may be attributed to the interactions with the active sites posed by the phenyl ring, as predicted by docking simulations. 

## 4. Experimental Design

### 4.1. Chemistry

All reactions were monitored by thin layer chromatography (TLC) on silica gel using 60 F254 aluminum sheets and were visualized under UV lamp at λ = 254 nm. The melting points were recorded and are uncorrected using a Stuart Scientific SMP1 device. The IR spectra were recorded on SHIMADZU FTIR-Affinity-1S spectrometer using KBr disks. The ^1^H NMR (400 MHz) and ^13^C NMR (100 MHz) spectra were recorded using a Bruker spectrometer (400 MHz) and TMS as an internal standard to calibrate the chemical shifts (δ) reported in ppm (See Supplementary Material Data). The microwave-assisted reactions were carried out in a programmable single-mode microwave reactor (CEM Discovery) that included a magnetic stirrer, pressure, temperature, and power controls, as well as a nitrogen cooling system. The reactor’s maximum operational pressure was 2106 Pa, and its power was set to 300 W. A CHN elemental analyzer was used to perform the elemental analysis.

#### 4.1.1. Synthesis and Characterization of ethyl 2-(4-phenyl-1H-1,2,3-triazol-1-yl)acetate (**3**)

*Conventional Method (CM):* To a solution of phenyl acetylene **1** (0.11 g, 1 mmoL) in *t*-BuOH (10 mL) was added a solution of copper sulfate (0.10 g, 0.4 mmoL) and sodium ascorbate (0.15 g, 0.75 mmoL) in water (10 mL). Then, ethyl-2-azidoacetate (**2**) (0.14 g, 1.1 mmoL) was added and the reaction mixture was stirred at room temperature for 6 h. TLC (hexane–ethyl acetate) was used to monitor the reaction, and after it was completed, iced water was added. The precipitate thus formed was collected by filtration, washed with a saturated solution of ammonium chloride, and recrystallized from ethanol to afford the targeted 1,2,3-triazole **3** with ester functionality.

*Microwave Method (MWI)*: In a closed borosilicate glass vessel fitted with a silicone cap, a mixture of phenyl acetylene **1** (0.11 g, 1 mmoL), copper sulfate (0.10 g, 0.4 mmoL), sodium ascorbate (0.15 g, 0.75 mmoL), ethyl-2-azidoacetate **2** (0.14 g, 1.1 mmoL), water (10 mL) and *t*-BuOH (10 mL) was irradiated by MWI for 3 min using a microwave reactor. The reaction mixture was then treated conventionally to get the same 1,2,3-triazole **3**.

#### 4.1.2. Synthesis and Characterization of 2-(4-phenyl-1H-1,2,3-triazol-1-yl)acetohydrazide (**4**)

*Conventional Method (CM):* A solution of ester **3** (0.23 g, 1 mmoL) in ethanol (20 mL) and hydrazine hydrate (0.1 g, 2 mmoL) was refluxed for 4 h. After cooling, ethanol was evacuated under reduced pressure, and the product formed was recrystallized from the ethanol yielding the 1,2,3-triazole-based acid hydrazide **4**.

*Microwave Method (MWI)*: A solution of ester **3** (0.12 g, 0.5 mmoL) in ethanol (10 mL) and hydrazine hydrate (0.05 g, 1 mmoL) was irradiated by MWI in a closed borosilicate glass vessel fitted with a silicone cap for 2 min. The reaction was conducted according to the conventional procedure stated earlier to produce the same 1,2,3-triazole **4**. 

#### 4.1.3. Synthesis of N-subtituted-2-(2-(4-phenyl-1H-1,2,3-triazol-1-yl)acetyl)hydrazine-1-carbothioamide **5**-**6**

*Conventional Method (CM):* A mixture of acid hydrazide **4** (2.1 g, 10 mmoL) in ethanol (20 mL) and the appropriate pheny/ethyl isothiocyanate (12 mmoL) was refluxed for 5–6 h. The precipitate produced after cooling was recovered by filtration and recrystallized from ethanol to get the desired acid thiosemicarbazides **5** and **6**.

*Microwave Method (MWI)*: A solution of the acid hydrazide **4** (0.1 g, 0.5 mmoL) in ethanol (10 mL) and appropriate isothiocayanate derivative (0.6 mmoL) was irradiated by MWI in a closed borosilicate glass vessel fitted with a silicone cap for 3–4 min. The same acid thiosemicarbazide derivatives **5** and **6** were produced by handling the reaction mixture, as indicated in the conventional procedure.

#### 4.1.4. Synthesis and Characterization of 4-substituted -3-((4-phenyl-1H-1,2,3-triazol-1-yl)methyl)-1H-1,2,4-triazole-5(4H)-thiones **7** and **8**

*Conventional Method (CM):* A solution of the acid thiosemicarbazide derivative **5** and/or **6** (1 mmoL) in aqueous sodium hydroxide 2 N (10 mL) was refluxed for 6 h. After cooling to room temperature, the solution was acidified with a diluted solution of hydrochloride acid to form the desired 1,2,4-triazoles **7** and/or **8** as precipitate and was filtered, washed with water, and recrystallized from ethanol.

*Microwave Method (MWI)*: A solution of acid thiosemicarbazide derivative **5** and/or **6** (1 mmoL) in aqueous sodium hydroxide 2 N (10 mL) was irradiated by MWI in a closed borosilicate glass vessel fitted with a silicone cap for 3–8 min using a microwave reactor. The reaction mixture was handled according to the conventional approach to give the same 1,2,4-triazoles **7** and/or **8**.

#### 4.1.5. 4-Phenyl-3-((4-phenyl-1H-1,2,3-triazol-1-yl)methyl)-1H-1,2,4-triazole-5(4H)-thione **7**

^1^H NMR (400 MHz, DMSO-*d*_6_): *δ*_ppm_ 14.11 (bs, 1 H, NH, D_2_O exchangeable), 8.27 (s, 1 H, H-5 of 1,2,3-triazole), 7.76–7.74 (m, 2 H, Ph-H), 7.48–7.42 (m, 5 H, Ph-H), 7.33 (bs, 3 H, Ph-H), 5.66 (s, 2 H, -CH_2_-). ^13^C NMR (100 MHz, DMSO-*d*_6_): *δ*_ppm_ 169.15 (C=S), 147.38 (C-3 of 1,2,4-triazole), 146.80 (C-4 of 1,2,3-triazole), 133.26, 130.79, 130.11, 129.86, 129.36, 128.46, 128.36, 125.65 (Ph-C), 122.55 (C-5 of 1,2,3-triazole), 44.99 (-CH_2_-). Calcd for C_17_H_14_N_6_S: C, 61.06; H, 4.22; N, 25.13. Found: C, 61.28; H, 4.39; N, 25.32. 

#### 4.1.6. 4-Ethyl-3-((4-phenyl-1H-1,2,3-triazol-1-yl)methyl)-1H-1,2,4-triazole-5(4H)-thione **8**

^1^H NMR (400 MHz, DMSO-*d*_6_): δ_ppm_ 13.93 (bs, 1 H, NH, D_2_O exchangeable), 8.71 (s, 1 H, H-5 of 1,2,3-triazole), 7.88 (d, 2 H, *J* = 8.0 Hz, Ph-H), 7.46–7.42 (m, 2 H, Ph-H), 7.34 (t, 1 H, *J* = 8.0 Hz, Ph-H), 5.93 (s, 2 H, -CH_2_-), 4.03 (q, 2 H, CH_2_CH_3_), 1.00 (t, 3 H, *J* = 8.0 Hz, CH_2_CH_3_). ^13^C NMR (100 MHz, DMSO-*d*_6_): *δ*_ppm_ 167.60 (C=S), 147.38 (C-3 of 1,2,4-triazole), 147.36 (C-4 of 1,2,3-triazole), 130.70, 129.42, 128.62, 125.73 (Ph-C), 122.68 (C-5 of 1,2,3-triazole), 44.55 (-CH_2_-), 39.23 (CH_2_CH_3_), 13.30 (CH_2_CH_3_). Calcd for C_13_H_14_N_6_S: C, 54.53; H, 4.93; N, 29.35. Found: C, 54.80; H, 4.79; N, 29.19.

#### 4.1.7. Synthesis and Characterization of Mannich Bases **9**–**16**

*Conventional Method (CM):* The appropriate secondary amine (1 mmoL) was added to a solution of triazole derivative **7** and/or **8** (1 and/or 2 mmoL) in ethanol (20 mL) in the presence of formaldehyde (1 and/or 2 mmoL), and then the reaction mixture was refluxed for 16–20 h (monitored using TLC). After completing the reaction, the solution was evaporated under reduced pressure, and the solid that resulted was recrystallized from ethanol to get the corresponding Mannich bases **9–16**.

*Microwave Method (MWI)*: A mixture of triazole derivative **7** and/or **8** (1 mmoL), appropriate secondary amine (1 and/or 2 mmoL), and formaldehyde (1 and/or 2 mmoL) in ethanol (10 mL) were placed in a sealed borosilicate glass vessel with a silicone cap and subjected to microwave radiation for 5–6 min. The reaction was treated conventionally to provide the same Mannich bases **9**–**16**.

NB: The reaction with piperazine as secondary amine required the use of 2 mmol of triazole **7**, **8** and formaldehyde.

#### 4.1.8. 4-Phenyl-3-((4-phenyl-1H-1,2,3-triazol-1-yl)methyl)-1-(piperidin-1-ylmethyl)-1H-1,2,4-triazole-5(4H)-thione (**9**)

^1^H NMR (400 MHz, DMSO-*d*_6_): *δ*_ppm_ 8.22 (s, 1 H, H-5 of 1,2,3-triazole), 7.73–7.71 (m, 2 H, Ph-H), 7.47–7.43 (m, 5 H, Ph-H), 7.33 (bs, 3 H, Ph-H), 5.71 (s, 2 H, 1,2,3-triazole-CH_2_-1,2,4-triazole), 5.07 (s, 2 H, NCH_2_N), 2.71 (bs, 4 H, 2× NCH_2_CH_2_CH_2_ piperidine), 1.50 (bs, 4 H, 2× NCH_2_CH_2_CH_2_ piperidine), 1.36 (bs, 2 H, NCH_2_CH_2_CH_2_ piperidine). ^13^C NMR (100 MHz, DMSO-*d*_6_): *δ*_ppm_ 169.88 (C=S), 146.72 (C-3 of 1,2,4-triazole), 145.92 (C-4 of 1,2,3-triazole), 133.70, 130.75, 130.16, 129.86, 129.36, 128.46, 128.34, 125.61 (Ph-C), 122.50 (C-5 of 1,2,3-triazole), 70.40 (NCH_2_N), 51.60 (2× NCH_2_CH_2_CH_2_ piperidine), 44.81 (1,2,3-triazole-CH_2_-1,2,4-triazole), 25.93 (2× NCH_2_CH_2_CH_2_ piperidine), 23.91 (NCH_2_CH_2_CH_2_ piperidine). Calcd for C_23_H_25_N_7_S: C, 64.01; H, 5.84; N, 22.72. Found: C, 64.33; H, 5.62; N, 22.45. 

#### 4.1.9. 4-Ethyl-3-((4-phenyl-1H-1,2,3-triazol-1-yl)methyl)-1-(piperidin-1-ylmethyl)-1H-1,2,4-triazole-5(4H)-thione (**10**)

^1^H NMR (400 MHz, DMSO-*d*_6_): *δ*_ppm_ 8.74 (s, 1 H, H-5 of 1,2,3-triazole), 7.88 (d, 2 H, Ph-H), 7.47–7.33 (m, 3 H, Ph-H), 5.99 (s, 2 H, 1,2,3-triazole-CH_2_-1,2,4-triazole), 4.98 (s, 2 H, NCH_2_N), 4.07 (q, 2 H, CH_2_CH_3_), 2.61 (bs, 4 H, 2× NCH_2_CH_2_CH_2_ piperidine), 1.45 (bs, 4 H, 2× NCH_2_CH_2_CH_2_ piperidine), 1.29 (bs, 2 H, NCH_2_CH_2_CH_2_ piperidine), 0.98 (t, 3 H, CH_2_CH_3_). ^13^C NMR (100 MHz, DMSO-*d*_6_): *δ*_ppm_ 168.37 (C=S), 147.34 (C-3 of 1,2,4-triazole), 145.99 (C-4 of 1,2,3-triazole), 130.70, 129.44, 128.46, 128.63, 125.71 (Ph-C), 122.71 (C-5 of 1,2,3-triazole), 70.00 (NCH_2_N), 51.58 (2× NCH_2_CH_2_CH_2_ piperidine), 44.35 (1,2,3-triazole-CH_2_-1,2,4-triazole), 39.30 (CH_2_CH_3_), 25.88 (2× NCH_2_CH_2_CH_2_ piperidine), 23.86 (NCH_2_CH_2_CH_2_ piperidine), 13.05 (CH_2_CH_3_). Calcd for C_19_H_25_N_7_S: C, 59.50; H, 6.57; N, 25.57. Found: C, 59.73; H, 6.29; N, 25.83.

#### 4.1.10. 1-(Morpholinomethyl)-4-phenyl-3-((4-phenyl-1H-1,2,3-triazol-1-yl)methyl)-1H-1,2,4-triazole-5(4H)-thione (**11**)

^1^H NMR (400 MHz, DMSO-*d*_6_): *δ*_ppm_ 8.23 (s, 1 H, H-5 of 1,2,3-triazole), 7.73–7.71 (m, 2 H, Ph-H), 7.47–7.42 (m, 5 H, Ph-H), 7.35 (bs, 3 H, Ph-H), 5.71 (s, 2 H, 1,2,3-triazole-CH_2_-1,2,4-triazole), 5.11 (s, 2 H, NCH_2_N), 3.59 (bs, 4 H, 2× NCH_2_CH_2_O morpholine), 2.75 (bs, 4 H, 2× NCH_2_CH_2_O morpholine). ^13^C NMR (100 MHz, DMSO-*d*_6_): *δ*_ppm_ 170.10 (C=S), 146.75 (C-3 of 1,2,4-triazole), 146.12 (C-4 of 1,2,3-triazole), 133.71, 130.77, 130.22, 129.89, 129.37, 128.48, 128.36, 125.64 (Ph-C), 122.60 (C-5 of 1,2,3-triazole), 69.44 (NCH_2_N), 66.55 (2× OCH_2_CH_2_N morpholine), 50.71 (2× OCH_2_CH_2_N morpholine), 44.82 (1,2,3-triazole-CH_2_-1,2,4-triazole). Calcd for C_22_H_23_N_7_OS: C, 60.95; H, 5.35; N, 22.62. Found: C, 60.69; H, 5.71; N, 22.84.

#### 4.1.11. 1-(Morpholinomethyl)-4-ethyl-3-((4-phenyl-1H-1,2,3-triazol-1-yl)methyl)-1H-1,2,4-triazole-5(4H)-thione (**12**)

^1^H NMR (400 MHz, DMSO-*d*_6_): *δ*_ppm_ 8.76 (s, 1 H, H-5 of 1,2,3-triazole), 7.88 (d, 2 H, Ph-H), 7.47–7.44 (m, 3 H, Ph-H), 7.37–7.35 (m, 1 H, Ph-H), 6.00 (s, 2 H, 1,2,3-triazole-CH_2_-1,2,4-triazole), 5.01 (s, 2 H, NCH_2_N), 4.10 (q, 2 H, CH_2_CH_3_), 3.53 (bs, 4 H, 2× NCH_2_CH_2_O morpholine), 2.64 (bs, 4 H, 2× NCH_2_CH_2_O morpholine), 1.00 (t, 3 H, CH_2_CH_3_). ^13^C NMR (100 MHz, DMSO-*d*_6_): *δ*_ppm_ 168.55 (C=S), 147.33 (C-3 of 1,2,4-triazole), 146.20 (C-4 of 1,2,3-triazole), 130.71, 129.44, 128.63, 125.72 (Ph-C), 122.75 (C-5 of 1,2,3-triazole), 69.07 (NCH_2_N), 66.48 (2× OCH_2_CH_2_N morpholine), 50.77 (2× OCH_2_CH_2_N morpholine), 44.37 (1,2,3-triazole-CH_2_-1,2,4-triazole), 39.30 (CH_2_CH_3_), 13.04 (CH_2_CH_3_). Calcd for C_18_H_23_N_7_OS: C, 56.08; H, 6.01; N, 25.43. Found: C, 56.31; H, 6.45; N, 25.66.

#### 4.1.12. 1-((4-Methylpiperazin-1-yl)methyl)-4-phenyl-3-((4-phenyl-1H-1,2,3-triazol-1-yl)methyl)-1H-1,2,4-triazole-5(4H)-thione (**13**)

^1^H NMR (400 MHz, DMSO-*d*_6_): *δ*_ppm_ 8.21 (s, 1 H, H-5 of 1,2,3-triazole), 7.73–7.71 (m, 2 H, Ph-H), 7.47–7.41 (m, 5 H, Ph-H), 7.33 (bs, 3 H, Ph-H), 5.71 (s, 2 H, 1,2,3-triazole-CH_2_-1,2,4-triazole), 5.10 (s, 2 H, NCH_2_N), 2.75 (bs, 4 H, 2× NCH_2_CH_2_NMe piperazine), 2.33 (bs, 4 H, 2× NCH_2_CH_2_NMe piperazine), 2.15 (s, 3 H, NCH_3_). ^13^C NMR (100 MHz, DMSO-*d*_6_): *δ*_ppm_ 169.95 (C=S), 146.72 (C-3 of 1,2,4-triazole), 146.00 (C-4 of 1,2,3-triazole), 133.70, 130.76, 130.21, 129.89, 129.37, 128.34, 125.64 (Ph-C), 122.66 (C-5 of 1,2,3-triazole), 69.27 (NCH_2_N), 54.95 (2× NCH_2_CH_2_NMe piperazine), 50.05 (2× NCH_2_CH_2_NMe piperazine), 46.17 (NCH_3_), 44.80 (1,2,3-triazole-CH_2_-1,2,4-triazole). Calcd for C_23_H_26_N_8_S: C, 61.86; H, 5.87; N, 25.09. Found: C, 61.69; H, 5.99; N, 25.38.

#### 4.1.13. 1-((4-Methylpiperazin-1-yl)methyl)-4-ethyl-3-((4-phenyl-1H-1,2,3-triazol-1-yl)methyl)-1H-1,2,4-triazole-5(4H)-thione (**14**)

^1^H NMR (400 MHz, DMSO-*d*_6_): *δ*_ppm_ 8.74 (s, 1 H, H-5 of 1,2,3-triazole), 7.88 (d, 2 H, Ph-H), 7.47–7.33 (m, 3 H, Ph-H), 5.99 (s, 2 H, 1,2,3-triazole-CH_2_-1,2,4-triazole), 5.02 (s, 2 H, NCH_2_N), 4.08 (q, 2 H, CH_2_CH_3_), 2.66 (bs, 4 H, 2× NCH_2_CH_2_NMe piperazine), 2.29 (bs, 4 H, 2× NCH_2_CH_2_NMe piperazine), 2.13 (s, 3 H, NCH_3_), 1.00 (t, 3 H, CH_2_CH_3_). ^13^C NMR (100 MHz, DMSO-*d*_6_): *δ*_ppm_ 168.45 (C=S), 147.35 (C-3 of 1,2,4-triazole), 146.07 (C-4 of 1,2,3-triazole), 130.71, 129.43, 128.63, 125.74 (Ph-C), 122.70 (C-5 of 1,2,3-triazole), 68.90 (NCH_2_N), 54.84 (2× NCH_2_CH_2_NMe piperazine), 49.98 (2× NCH_2_CH_2_NMe piperazine), 46.05 (NCH_3_), 44.35 (1,2,3-triazole-CH_2_-1,2,4-triazole), 39.31 (CH_2_CH_3_), 13.06 (CH_2_CH_3_). Calcd for C_19_H_26_N_8_S: C, 57.26; H, 6.58; N, 28.12. Found: C, 57.54; H, 6.33; N, 28.46.

#### 4.1.14. 1,1′-(piperazine-1,4-diylbis(methylene))bis(4-phenyl-3-((4-phenyl-1H-1,2,3-triazol-1-yl)methyl)-1H-1,2,4-triazole-5(4H)-thione) (**15**)

^1^H NMR (400 MHz, DMSO-*d*_6_): *δ*_ppm_ 8.46, 8.28 (2 s, 2 H, 2× H-5 of 1,2,3-triazole), 7.87–7.85 (m, 2 H, Ph-H), 7.74–7.73 (m, 2 H, Ph-H), 7.48–7.42 (m, 10 H, Ph-H), 7.34–7.31 (m, 6 H, Ph-H), 5.71, 5.68 (2 s, 4 H, 2× 1,2,3-triazole-CH_2_-1,2,4-triazole), 4.92 (s, 4 H, 2× NCH_2_N), 3.03–3.00 (m, 4 H, 2× NCH_2_CH_2_N piperazine), 2.19 (bs, 4 H, 2× NCH_2_CH_2_N piperazine). ^13^C NMR (100 MHz, DMSO-*d*_6_): *δ*_ppm_ 169.67, 169.11 (2× C=S), 147.37, 146.79 (2× C-3 of 1,2,4-triazole), 146.30, 146.11 (2× C-4 of 1,2,3-triazole), 133.54, 133.26, 131.58, 130.76, 130.73, 130.28, 130.10, 129.94, 129.86, 129.37, 129.34, 128.51, 128.36, 128.26, 128.05, 125.65, 125.48 (Ph-C), 123.01, 122.61 (2× C-5 of 1,2,3-triazole), 53.59 (2× NCH_2_N), 51.68 (2× NCH_2_CH_2_N piperazine), 45.87 (2× NCH_2_CH_2_N piperazine), 42.91 (2× 1,2,3-triazole-CH_2_-1,2,4-triazole). Calcd for C_40_H_38_N_14_S_2_: C, 61.68; H, 4.92; N, 25.17. Found: C, 61.81; H, 4.79; N, 25.36.

#### 4.1.15. 1,1′-(piperazine-1,4-diylbis(methylene))bis(4-ethyl-3-((4-phenyl-1H-1,2,3-triazol-1-yl)methyl)-1H-1,2,4-triazole-5(4H)-thione) (**16**)

^1^H NMR (400 MHz, DMSO-*d*_6_): *δ*_ppm_ 8.73 (s, 2 H, 2× H-5 of 1,2,3-triazole), 7.87–7.86 (m, 4 H, Ph-H), 7.47–7.44 (m, 4 H, Ph-H), 7.37–7.35 (m, 2 H, Ph-H), 5.97 (s, 4 H, 2× 1,2,3-triazole-CH_2_-1,2,4-triazole), 4.98 (s, 4 H, 2× NCH_2_N), 4.06–4.05 (m, 4 H, 2× CH_2_CH_3_), 2.64 (bs, 8 H, 4 X CH_2_ piperazine), 0.99 (bs, 3 H, CH_2_CH_3_). ^13^C NMR (100 MHz, DMSO-*d*_6_): *δ*_ppm_ 168.40 (2× C=S), 147.34 (2× C-3 of 1,2,4-triazole), 146.06 (2× C-4 of 1,2,3-triazole), 130.71, 129.44, 128.65, 125.74 (Ph-C), 122.71 (2× C-5 of 1,2,3-triazole), 68.93 (2× NCH_2_N), 50.14 (CH_2_ piperazine), 44.35 (2× 1,2,3-triazole-CH_2_-1,2,4-triazole), 39.33 (2× CH_2_CH_3_), 13.08 (2× CH_2_CH_3_). Calcd for C_32_H_38_N_14_S_2_: C, 56.28; H, 5.61; N, 28.72. Found: C, 56.47; H, 5.82; N, 28.88.

### 4.2. Biology

#### 4.2.1. Cytotoxicity Screening 

Cytotoxicity of the studied compounds was assayed on normal human lung fibroblasts (Wi-38) and cancer cells (MDA-MB 231, and Caco-2), as previously described, via MTT assay [57,59].

#### 4.2.2. Quantitative Real-Time PCR Analysis of p53 and Cyclin D 

Total RNAs of the untreated and treated cancer cells were extracted and reversed transcribed to cDNA using the Gene JET RNA Purification Kit (Thermo Scientific, Waltham, MA, USA) and cDNA Synthesis Kit (Thermo Scientific, USA), respectively. Real-time PCR was performed using SYBR green master mix and specific primers (Forward/Reverse) were 5′-ATGTTTTGCCAACTGGCCAAG-3/5′-TGAGCAGCGCTCATGGTG-3′ and 5′-TACTCTGGCGCAGAAATTAGGTC-3′/5′-CTGTCTCGGAGCTCGTCTATTTG-3′ for p53 and cyclin D, respectively.

#### 4.2.3. MMP-2/9 Inhibition Assays 

MMP-2/9 inhibitory activities were detected for the studied compounds according to the inhibitor screening assay colorimetric kit (Abcam, Catalog No. ab139446 for MMP-2 and ab139448 for MMP-9) instructions. Briefly, the studied compounds and the reference inhibitor (NNGH) at serial concentrations were added to assay buffer and MMP, then incubated for 60 min at 37 °C. After that, the chromogenic substrate was added, and the absorbance (ΔA) was recorded per min for 10 min at 412 nm. The inhibition percentage of MMP-9 for each compound was calculated as follows:

100-([velocity (ΔA/min) of compound/velocity (ΔA/min) of control (no inhibitor added)] * 100). IC_50_ values were computed using Graphpad Prism. 

#### 4.2.4. Molecular Modeling

##### Docking Simulations

Docking simulations were conducted employing MOE 2015.10 [73]. Briefly, the coordinates of MMP-2 and -9 active sites were retrieved from the protein data bank. Unwanted molecules were deleted. The protein structures were prepared via the default “QuickPrep” module settings. The studied derivatives were subjected to the MMFF94x forcefield and gradient: 0.05 for energy minimization after adding hydrogens and applying partial charges. The binding sites encompassing key amino acid residues were located via the ‘Site Finder’ feature. Rigid docking was simulated by MOE employing the Triangle Matcher placement method, Rescoring 1: London dG, Refinement: Forcefield, and Rescoring 2: Affinity dG. After docking, the conformations were visualized and selected based on the protein–ligand interactions and docking scores. 

##### ADMET and Drug-likeness Prediction

ADME and drug-likeness parameters were computed by *SwissADME* [76] and *Pre-ADMET* calculator [77]. Toxicity (LD_50_) was predicted employing PROTOX [78]. 

#### 4.2.5. Data Analysis 

Biological data were expressed as mean ± SEM. Statistical significance was estimated by the multiple comparisons Tukey post-hoc analysis of variance (ANOVA) employing SPSS16 and differences were considered significant at *p* < 0.05. IC_50_ values were computed using Graphpad Prism.

## 5. Conclusions

The current study portrays the design, synthesis, and evaluation of non-hydroxamate MMP-2/9 inhibitors based on tethered isomeric triazoles Mannich bases with varying substituents and lengths. The rationale design relied on mimicking the non-hydroxamate inhibitors structural outlines. Among the synthesized series, the bis-Mannich bases **15** and **16** were superior to the reference chemotherapy 5-fluorouracil regarding safety, selectivity, and anticancer activity (submicromolar IC_50_) against MDA-MB 231 and Caco-2 cells. In the studied cancers, **15** and **16** upregulated p53, where **15** exhibited higher p53 induction potential (up to 5.6-fold) than that recorded for **16** (up to 2.9-fold). Additionally, they suppressed cyclin D expression (down to 0.2-fold) in the treated cancer cell lines, therefore inducing apoptosis. Moreover, **15** was also superior to **16** in terms of cyclin D suppression leading to 0.23- and 0.38-fold relative decrease in cyclin D in the treated breast and colon cancer cells, respectively, compared to 0.49- and 0.87-fold, respectively, in the **16**-treated cancer cells. Both compounds exhibited promising MMP-2/9 inhibitory activities, comparable to the reference MMP inhibitor NNGH, at their anticancer IC_50_ doses. Moreover, **15** was 4-fold more active against MMP-9 than NNGH, with 3.27-fold selectivity over MMP-2. The less potent derivative **16** was comparable to NNGH. On the other hand, **16** was 1.2-fold more active than **15** against MMP-2. Docking simulations of **15** and **16** into MMP-2 and -9 catalytic domains predicted suitable fitting of the compounds into the active sites at the proximity of S1′ pockets with considerable key interactions. In silico ADMET and drug-likeness prediction recorded acceptable metrics. 

## Figures and Tables

**Table 1:** Comparison between the conventional method (CM) and microwave irradiation (MW) in the synthesis of compounds **3**–**16**.

Compound	[Ref.] Mp (°C)	Time		Yield (%)	
		CM (hr)	MW (min)	CM	MW
**3**	102–103 [60] 104	6	3	91	98
**4**	200–201 [61] 203–205	4	2	89	97
**5**	241–242 [62] 238–240	4	3	88	96
**6**	212–214 [62] 224–226	4	3	86	95
**7**	267–269 [63] 269–270	6	4	91	97
**8**	218–219	6	4	90	96
**9**	188–189	16	5	85	92
**10**	160–161	16	5	86	90
**11**	204–205	16	5	85	94
**12**	178–179	16	5	84	94
**13**	228–229	20	6	83	96
**14**	194–195	20	6	83	96
**15**	293–294	20	6	81	92
**16**	259–260	20	6	82	92

**Table 2:** Cytotoxicity and selectivity index (SI) values of the Mannich bases **9**–**16.**

Compound No.	Wi-38		MDA-MB 231		Caco-2	
	EC_100_ (µM) *	EC_50_ (µM)	IC_50_ (µM)	SI	IC_50_ (µM)	SI
**9**	0.638 ± 0.029	1.324 ± 0.024	1.733 ± 0.068	0.763	2.292 ± 0.084	0.577
**10**	1.460 ± 0.026	2.250 ± 0.048	1.388 ± 0.082	1.621	1.628 ± 0.006	1.382
**11**	0.653 ± 0.036	1.755 ± 0.039	2.354 ± 0.022	0.745	2.121 ± 0.009	0.827
**12**	0.984 ± 0.034	2.745 ± 0.054	1.146 ± 0.093	2.395	3.313 ± 0.110	0.828
**13**	0.591 ± 0.048	1.145 ± 0.042	0.733 ± 0.056	1.562	0.609 ± 0.016	1.880
**14**	0.850 ± 0.036	1.721 ± 0.056	2.037 ± 0.044	0.844	1.727 ± 0.028	0.996
**15**	0.804 ± 0.016	1.089 ± 0.007	0.306 ± 0.010	3.558	0.264 ± 0.001	4.125
**16**	0.463 ± 0.034	0.779 ± 0.033	0.404 ± 0.015	1.928	0.390 ± 0.001	1.997
**5-Fluorouracil**	1.067 ± 0.002	2.915 ± 0.052	21.019 ± 0.032	-	0.437 ± 0.016	-

* All values are expressed are mean ± SEM.

**Table 3:** Inhibitory profiles of **15** and **16** against MMP-2 and -9.

Compound No.	MMP-2		MMP-9		MMP-2/MMP-9 Selectivity
	% Inhibition at Anticancer IC_50_	IC_50_ (μM)	% Inhibition at Anticancer IC_50_	IC_50_ (μM)	
**15**	46.015 ± 1.290	0.468	64.936 ± 1.603	0.143	3.27
**16**	54.630 ± 0.997	0.376	55.090 ± 2.053	0.387	0.97
**NNGH**	60.855 ± 1.714	0.298	56.463 ± 1.990	0.349	0.85

Values are expressed as mean ± SEM.

**Table 4:** In silico physicochemical properties, ADMET, and drug-likeness.

Cpd No.	Physiochemical Parameters							ADMET								Drug-Likeness		
	LogP ^a^	MW ^b^	HBA ^c^	HBD ^d^	NROTB ^e^	TPSA ^f^	S ^g^	HIA ^h^	PPB ^i^	BBB ^j^	Caco2 ^k^	MDCK ^l^	CYP3A4 Inhibitor	CYP2D6 Inhibitor	LD_50_^m^	Lipinski ^n^	Veber ^o^	Egan ^p^
**15**	4.02	778.95	8	0	12	177.58	5.57 × 10^−6^	98.10	100	1.88	34.90	1.49	Yes	Non	1000	1 violation: MW > 500	2 violations: Rotors > 10, TPSA > 140	1 violation: TPSA > 131.6
**16**	2.44	682.87	8	0	12	177.58	0.004	99.19	93.48	0.515	32.69	0.04	Yes	Non	1000	1 violation: MW > 500	2 violations: Rotors > 10, TPSA > 140	1 violation: TPSA > 131.6

^a^ Log P: logarithm of compound partition coefficient between n-octanol and water. ^b^ M.Wt: molecular weight. ^c^ HBA: number of hydrogen bond acceptors. ^d^ HBD: number of hydrogen bond donors. ^e^ NROTB: number of rotatable bonds. ^f^ TPSA: polar surface area. Drug-like TPSA < 140–150 A^2^. ^g^ S: aqueous solubility (mg/L). ^h^ HIA: human intestinal absorption. HIA values < 20% (poorly absorbed), values ≈ 20–70% (moderately absorbed) and values > 70% (well absorbed) [82]. ^i^ PPB: plasma protein binding. PPB values < 90% (poorly bound) and values > 90% (strongly bound) [83]. ^j^ BBB: blood–brain barrier penetration. BBB values < 0.1 (low CNS absorption), values ≈ 0.1–2 (medium CNS absorption) and values > 2 (high CNS absorption) [84]. ^k^ Caco2: permeability through cells derived from human colon adenocarcinoma. P_Caco2_ values < 4 nm/sec (low permeability), values ≈ 4–70 nm/sec (medium permeability) and values > 70 nm/sec (high permeability) [85,86,87]. ^l^ MDCK: permeability through Madin-Darby Canin kidney cells. P_MDCK_ values < 25 nm/sec (low permeability), values ≈ 25–500 nm/sec (medium permeability) and values > 500 nm/sec (high permeability) [86]. ^m^ LD_50_: the median lethal dose (mg/Kg). Toxicity classes according to GHS are: Class I: fatal if swallowed (LD50 ≤ 5), Class II: fatal if swallowed (5 < LD50 ≤ 50), Class III: toxic if swallowed (50 < LD50 ≤ 300), Class IV: harmful if swallowed (300 < LD50 ≤ 2000), Class V: may be harmful if swallowed (2000 < LD50 ≤ 5000), and Class VI: non-toxic (LD50 > 5000) [78]. ^n^ Lipinski rule: log P ≤ 5, M.Wt ≤ 500 Da, HBA ≤ 10 and HBD ≤ 5 [79]. ^o^ Veber rule: NROTB ≤ 10 and TPSA ≤ 140 [80]. ^p^ Egan’s rule: log P ≤ 5.88, TPSA ≤ 131.6 [81].

## Data Availability

All compounds are available at Najate lab.

## Supplementary Materials

The following are available online at www.mdpi.com/article/10.3390/ijms221910324/s1.
