# Supplementary material for: Halting Tumor Progression via Novel Non-Hydroxamate Triazole-Based Mannich Bases MMP-2/9 Inhibitors; Design, Microwave-Assisted Synthesis, and Biological Evaluation
Source: Int J Mol Sci. 2021 Sep 25;22(19):10324. doi: 10.3390/ijms221910324 (PMC8508768; doi:10.3390/ijms221910324)
Supplement: Supplementary file 1 [file ijms-22-10324-s001.zip › ijms-1369206-supplementary.pdf]

Supplementary Information

Halting tumor progression *via* novel non-hyroxamate triazole-based Mannich bases MMP-2/9 inhibitors; rationale design, microwave assisted synthesis and biological evaluation

Fawzia Faleh Albelwi [1], Mohamed Teleb [2], Marwa M. Abu-Serie [3], Mohamed Nabil Abd Al Moaty [4], Mai S. Alsubaie [4], Mohamed A. Zakaria [4], Yeldez El Kilany [4], Mohamed Reda Aouad[1], Mohamed Hagar[4,*] and Nadjet Rezki[1,*]

1. *Department of Chemistry, Faculty of Science, Taibah University, Al-Madinah Al-Munawarah 30002, Saudi Arabia*
2. Department of Pharmaceutical Chemistry, Faculty of Pharmacy, Alexandria University, Alexandria, 21521, Egypt.
3. Medical Biotechnology Department, Genetic Engineering and Biotechnology Research Institute, City of Scientific Research and Technological Applications (SRTA-City), Egypt
4. *Chemistry Department, Faculty of Science, Alexandria University, Alexandria 21321, Egypt*

*Correspondence: M.H.: mohamedhaggar@gmail.com; N.R.: nadjetrezki@yahoo.fr

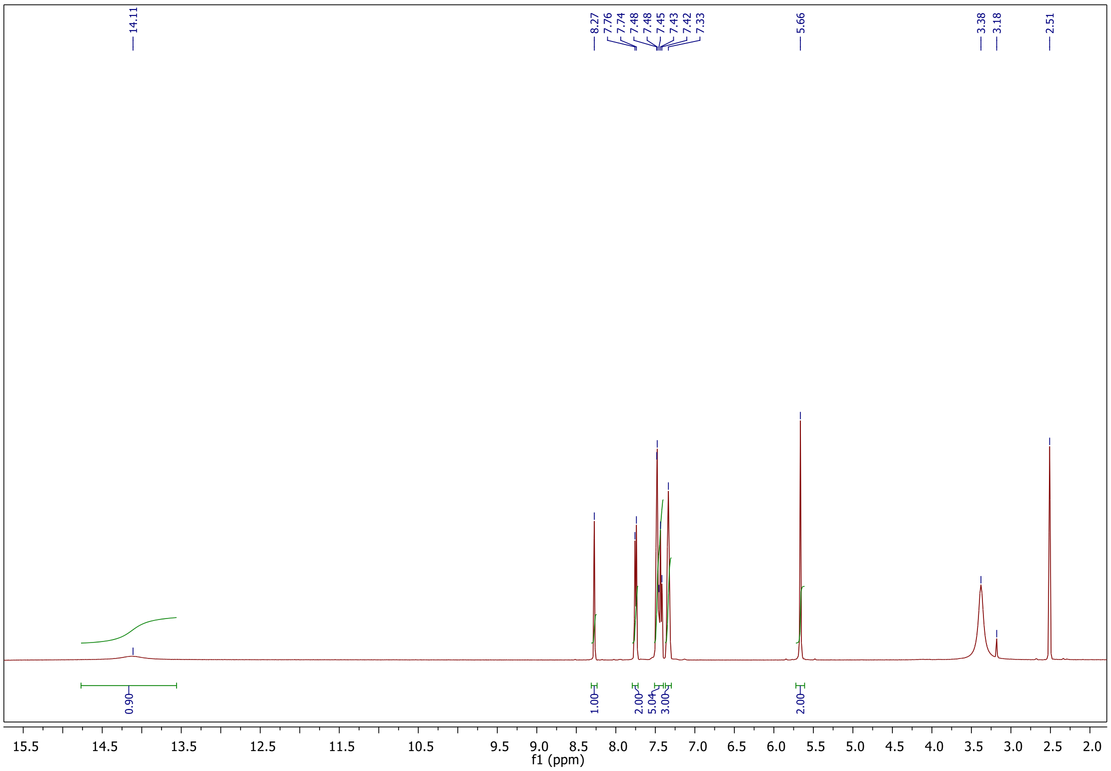

Figure S1. ^{1}H-NMR Spectrum of compound **7** (DMSO-d_6, 400 MHz).

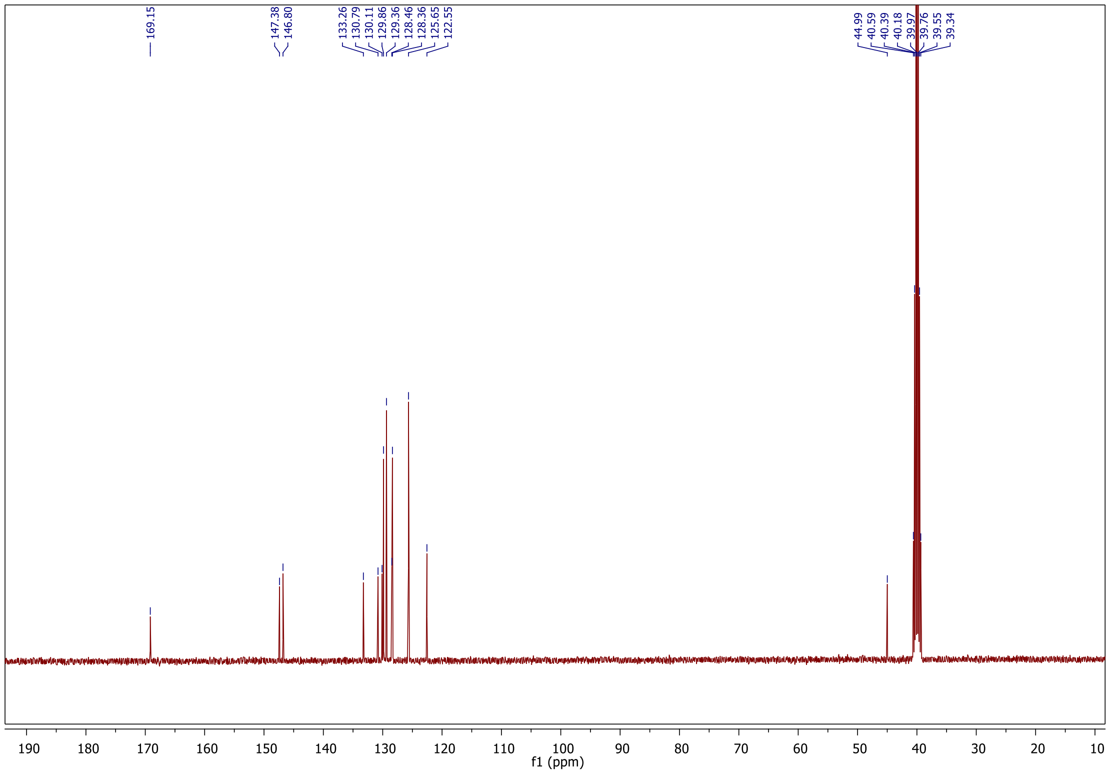

Figure S2. ^{13}C-NMR Spectrum of compound **7** (DMSO-d_6, 100 MHz).

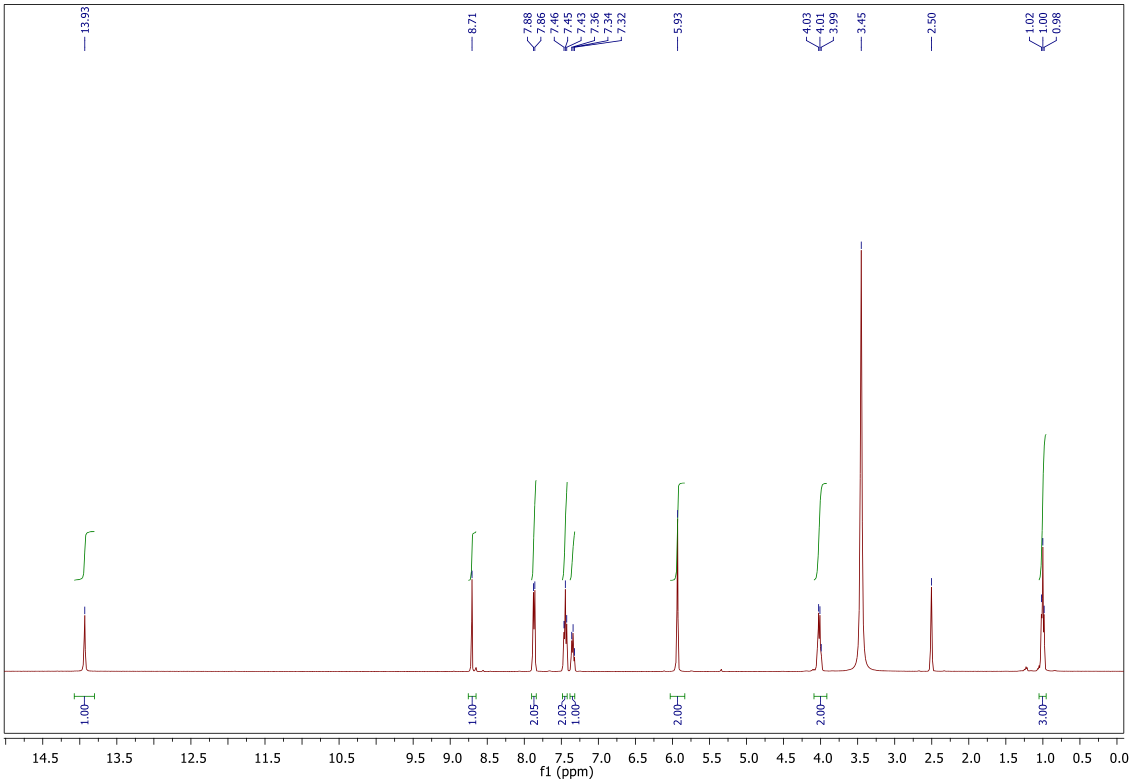

Figure S3. ^{1}H-NMR Spectrum of compound **8** (DMSO-d_6, 400 MHz).

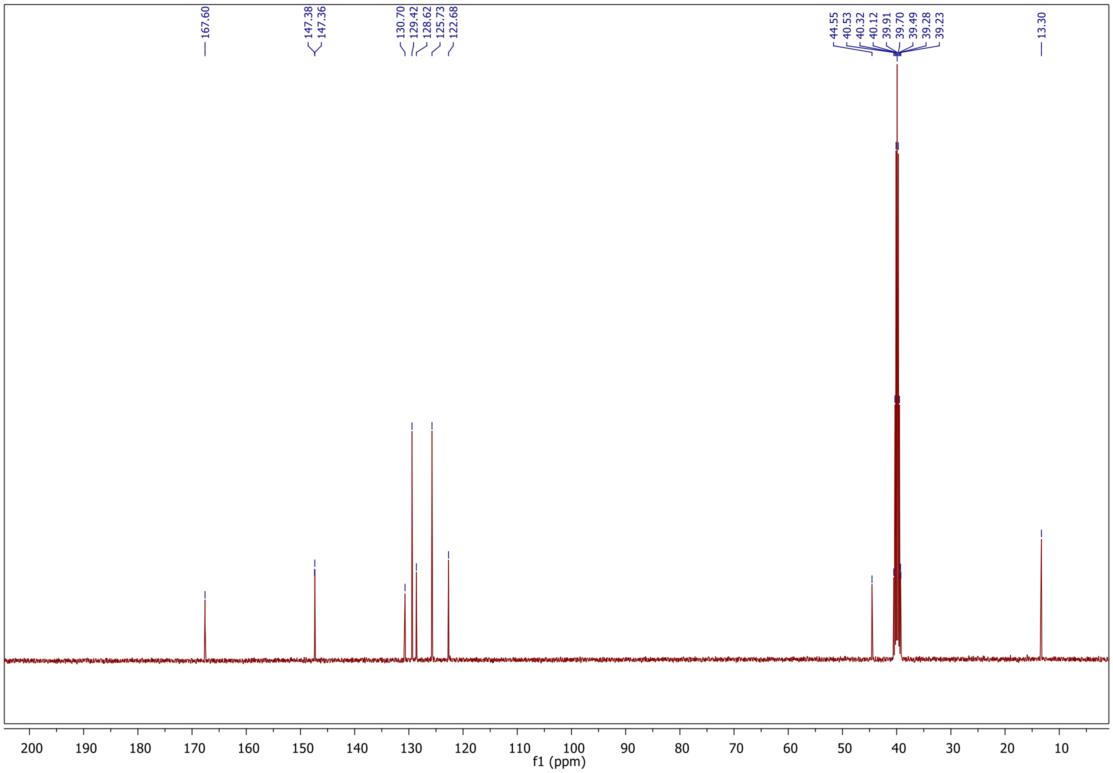

Figure S4. ^{13}C-NMR Spectrum of compound **8** (DMSO-d_6, 100 MHz).

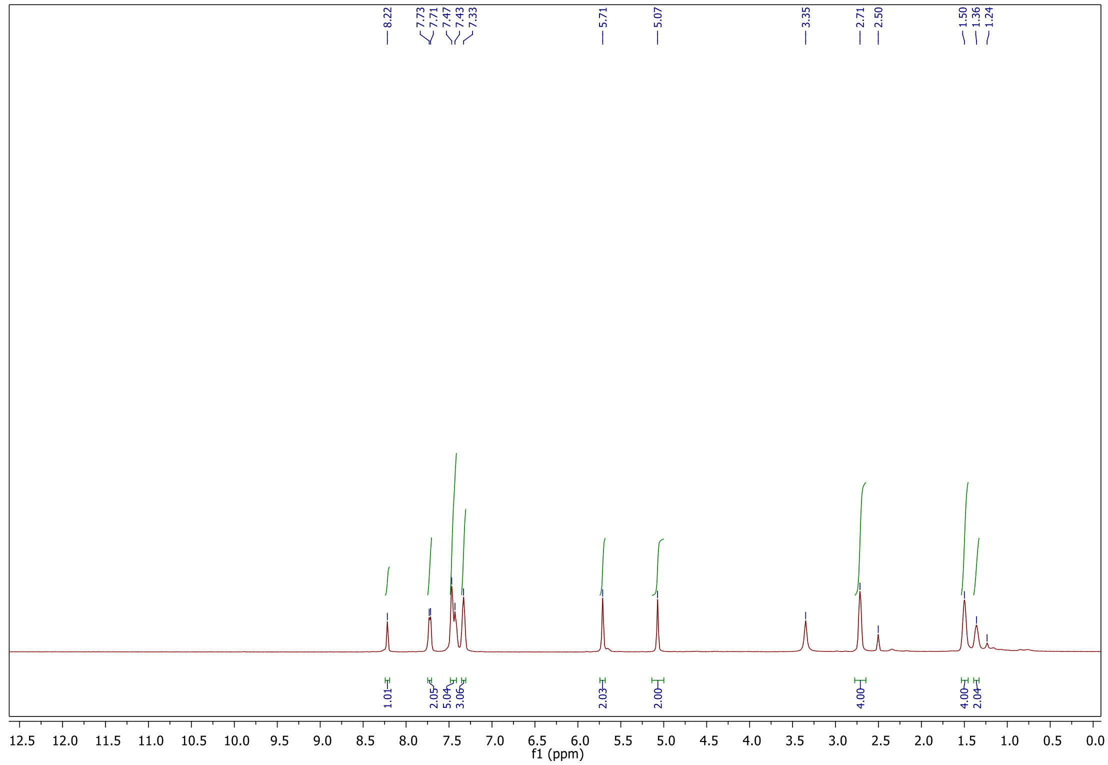

Figure S5. ^{1}H-NMR Spectrum of compound **9** (DMSO-d_6, 400 MHz).

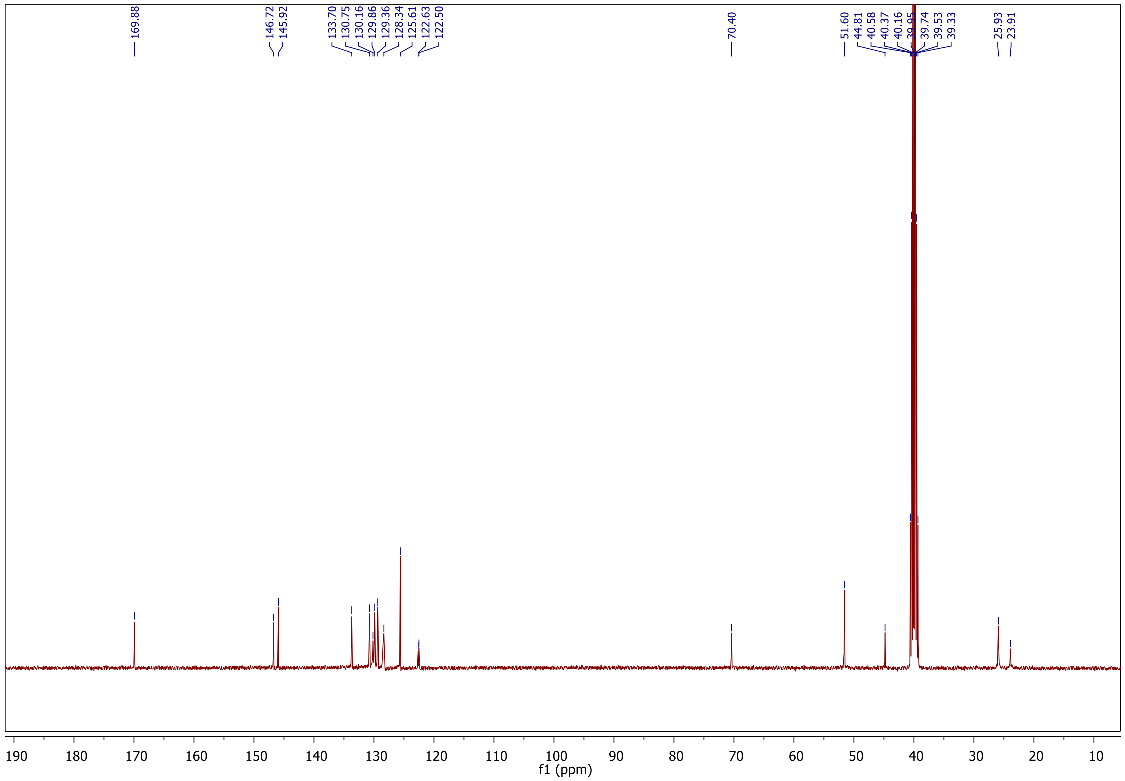

Figure S6. ^{13}C-NMR Spectrum of compound **9** (DMSO-d_6, 100 MHz).

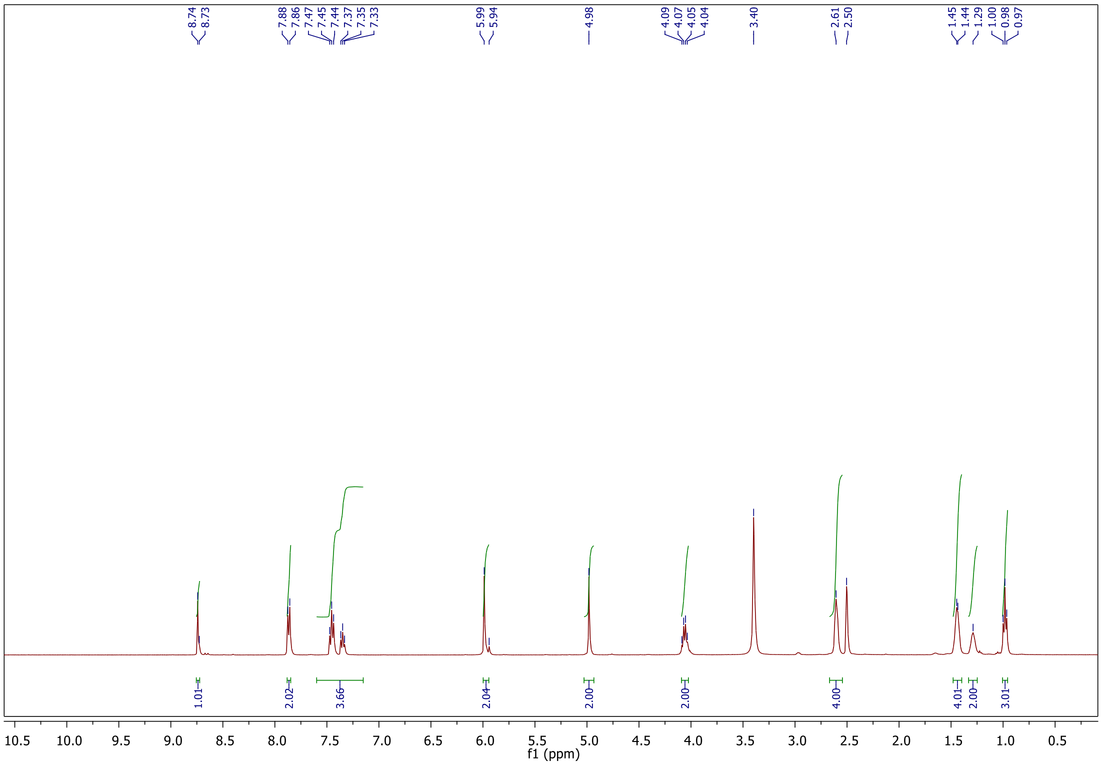

Figure S7. ^{1}H-NMR Spectrum of compound **10** (DMSO-d_6, 400 MHz).

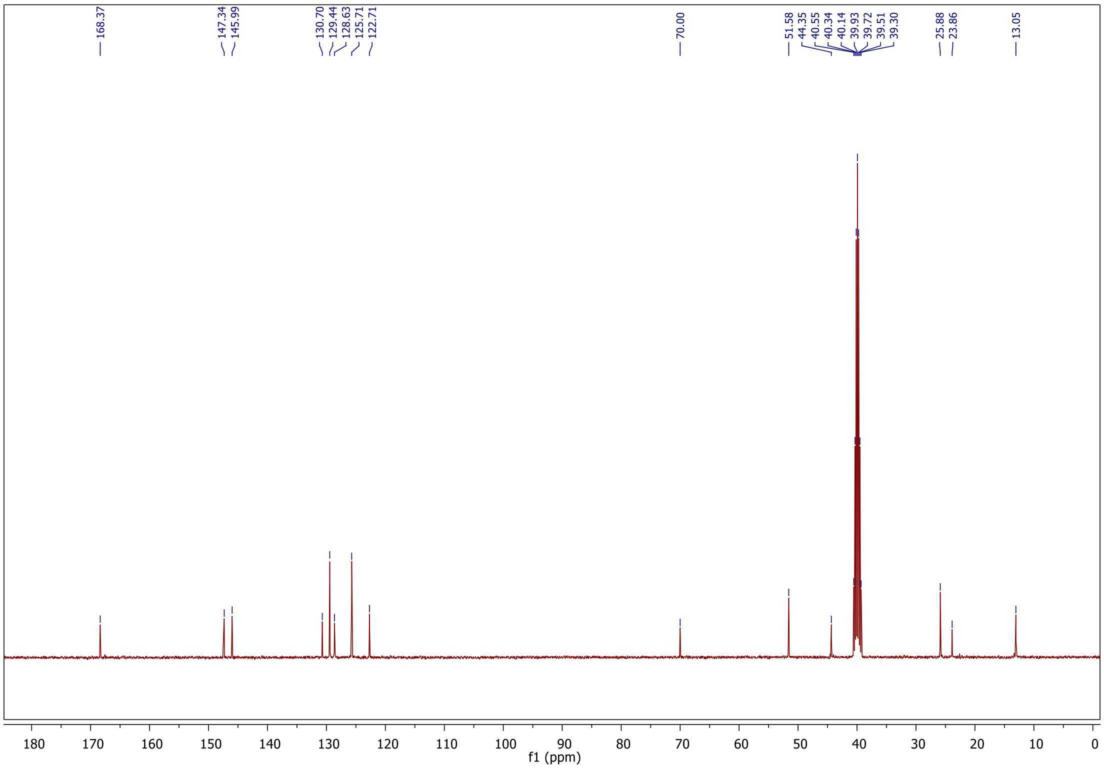

Figure S8. ^{13}C-NMR Spectrum of compound **10** (DMSO-d_6, 100 MHz).

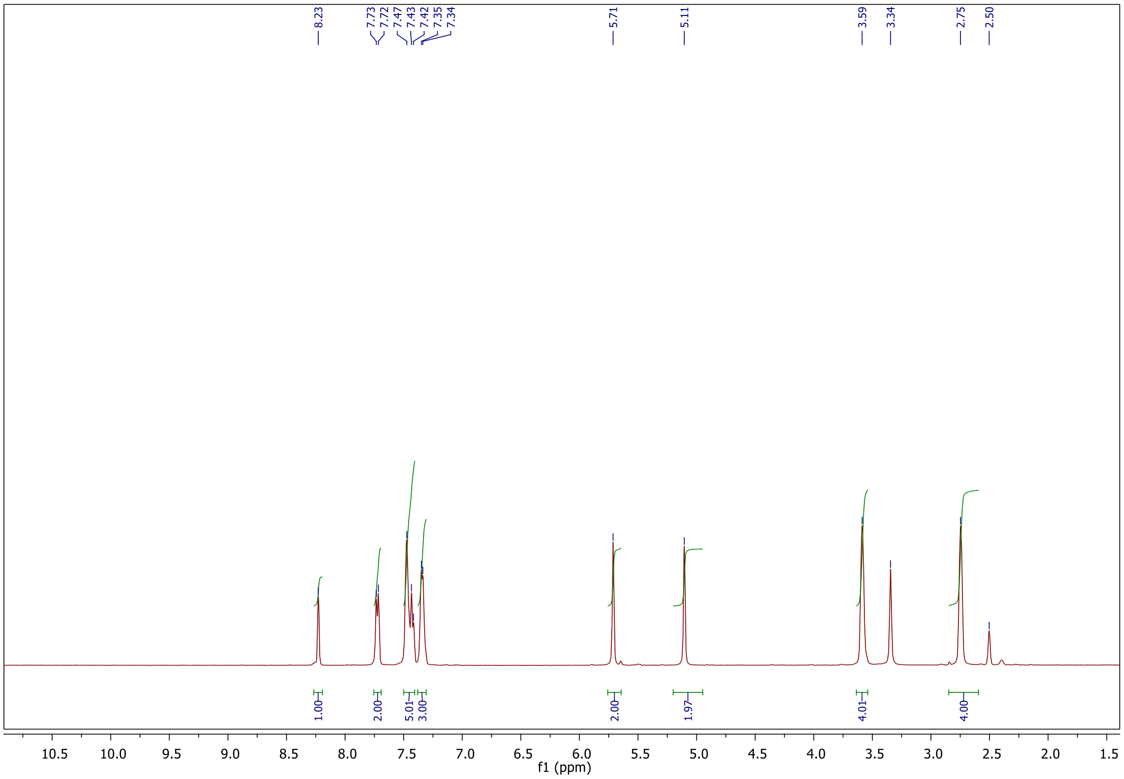

Figure S9. ^{1}H-NMR Spectrum of compound **11** (DMSO-d_6, 400 MHz).

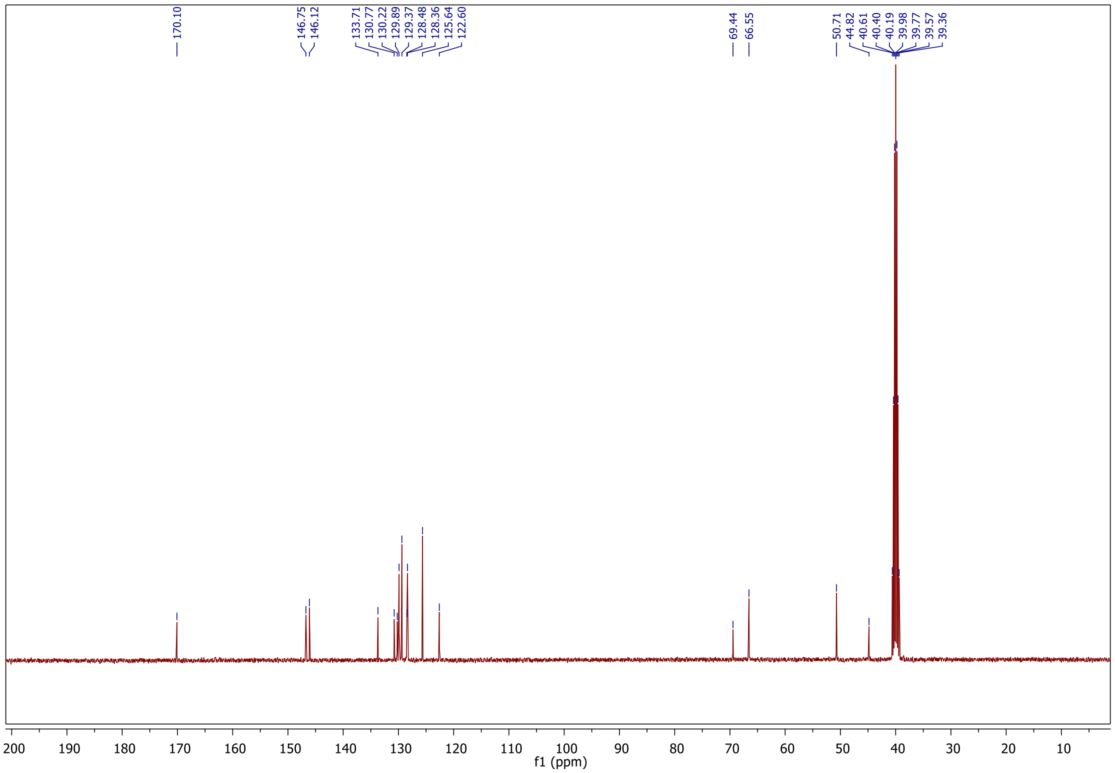

Figure S10. ^{13}C-NMR Spectrum of compound **11** (DMSO-d_6, 100 MHz).

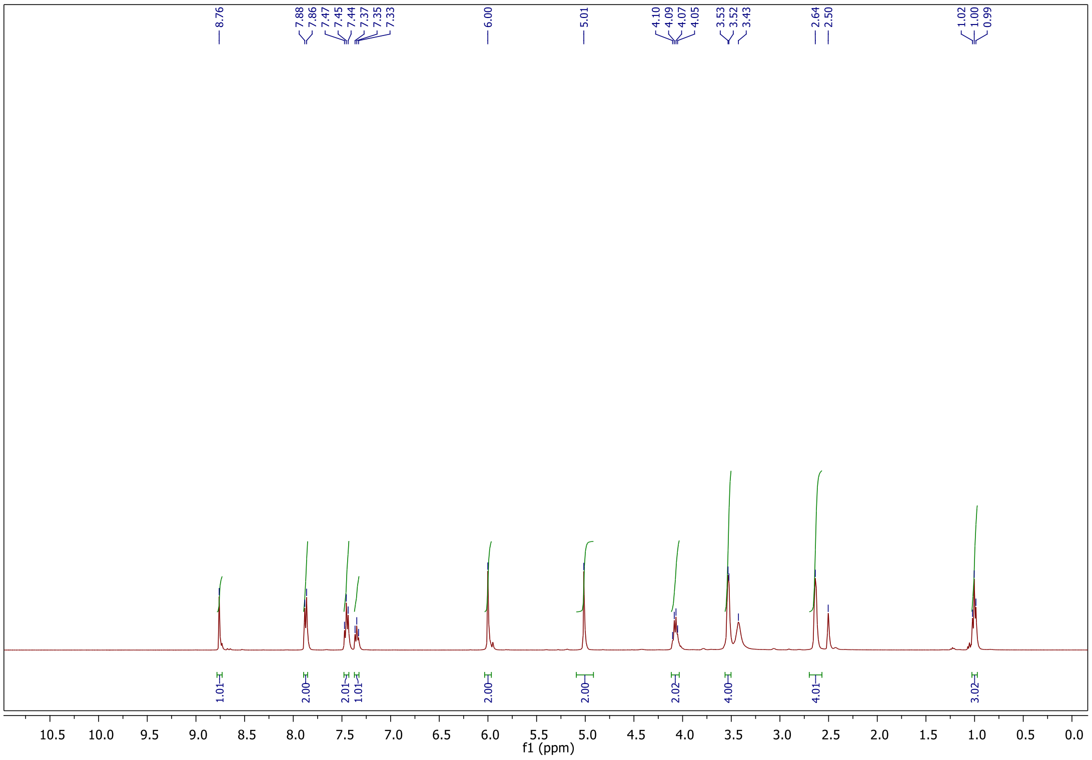

Figure S11. ^{1}H-NMR Spectrum of compound **12** (DMSO-d_6, 400 MHz).

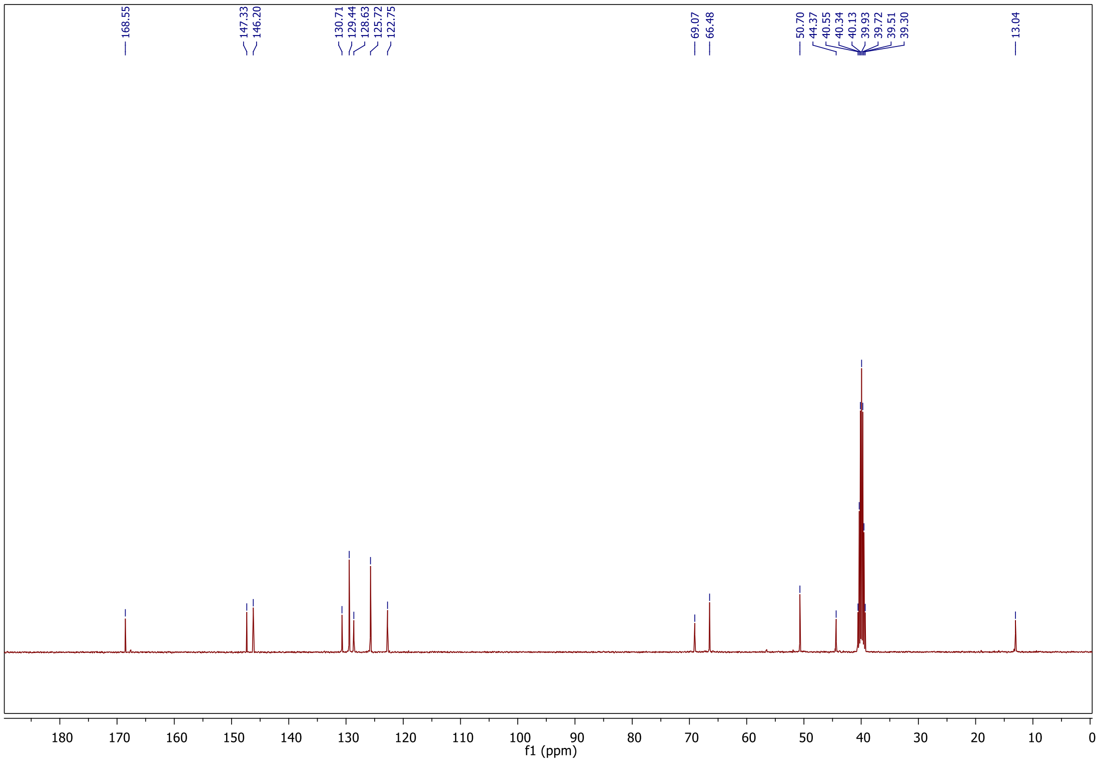

Figure S12. ^{13}C-NMR Spectrum of compound **12** (DMSO-d_6, 100 MHz).

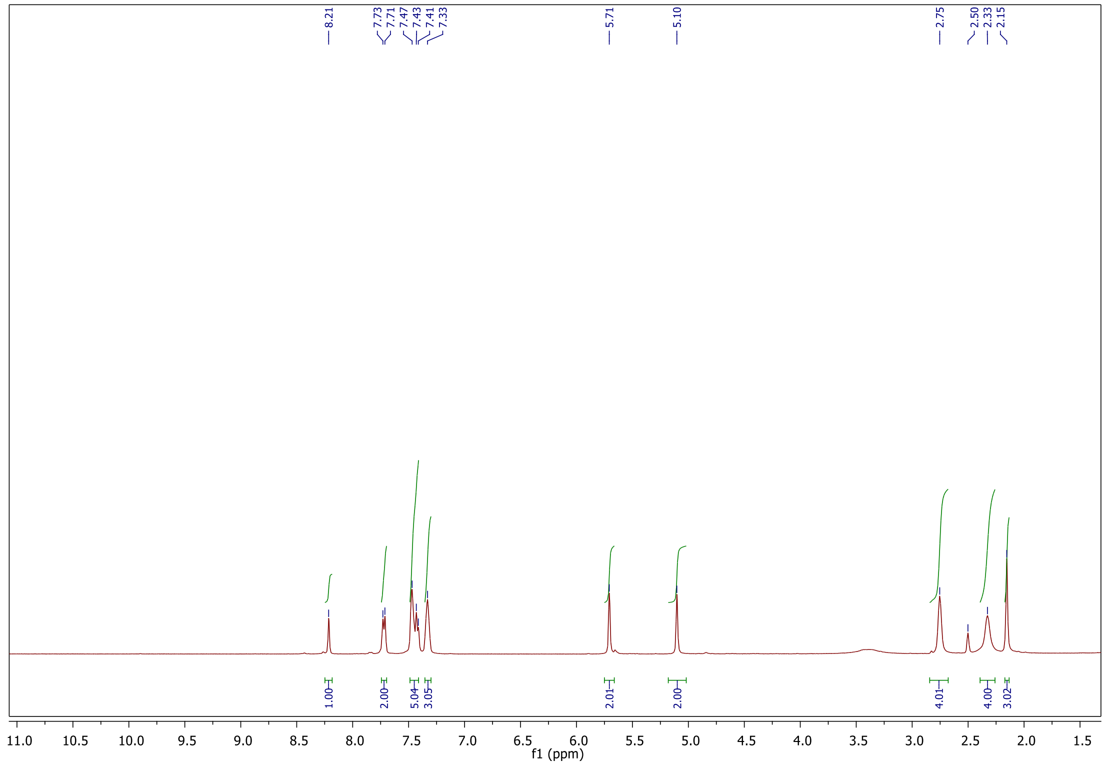

Figure S13. ^{1}H-NMR Spectrum of compound **13** (DMSO-d_6, 400 MHz).

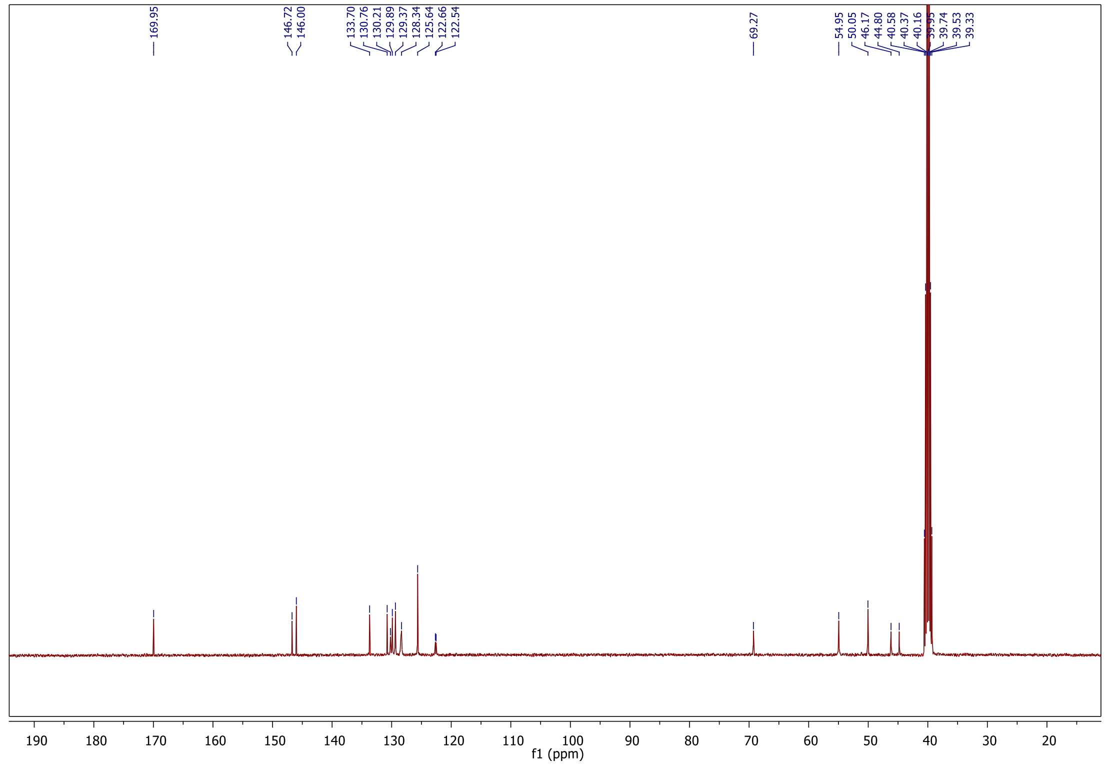

Figure S14. ^{13}C-NMR Spectrum of compound **13** (DMSO-d_6, 100 MHz).

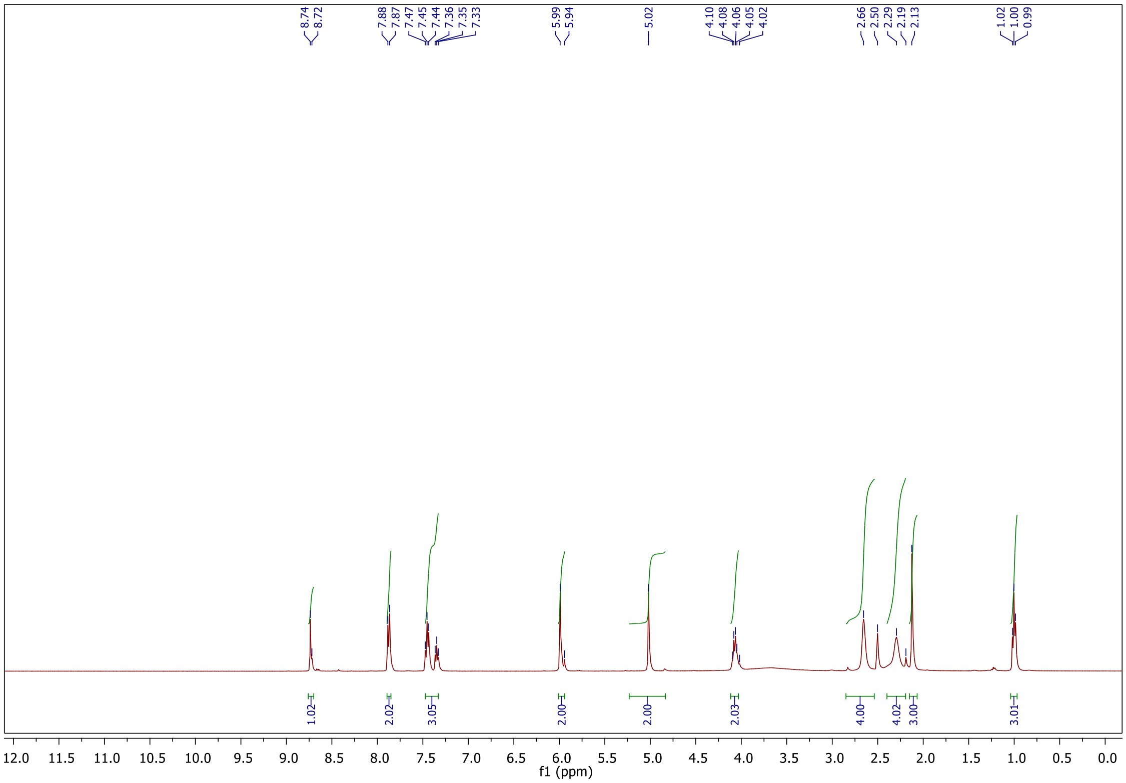

Figure S15. ^{1}H-NMR Spectrum of compound **14** (DMSO-*d*$_6$, 400 MHz).

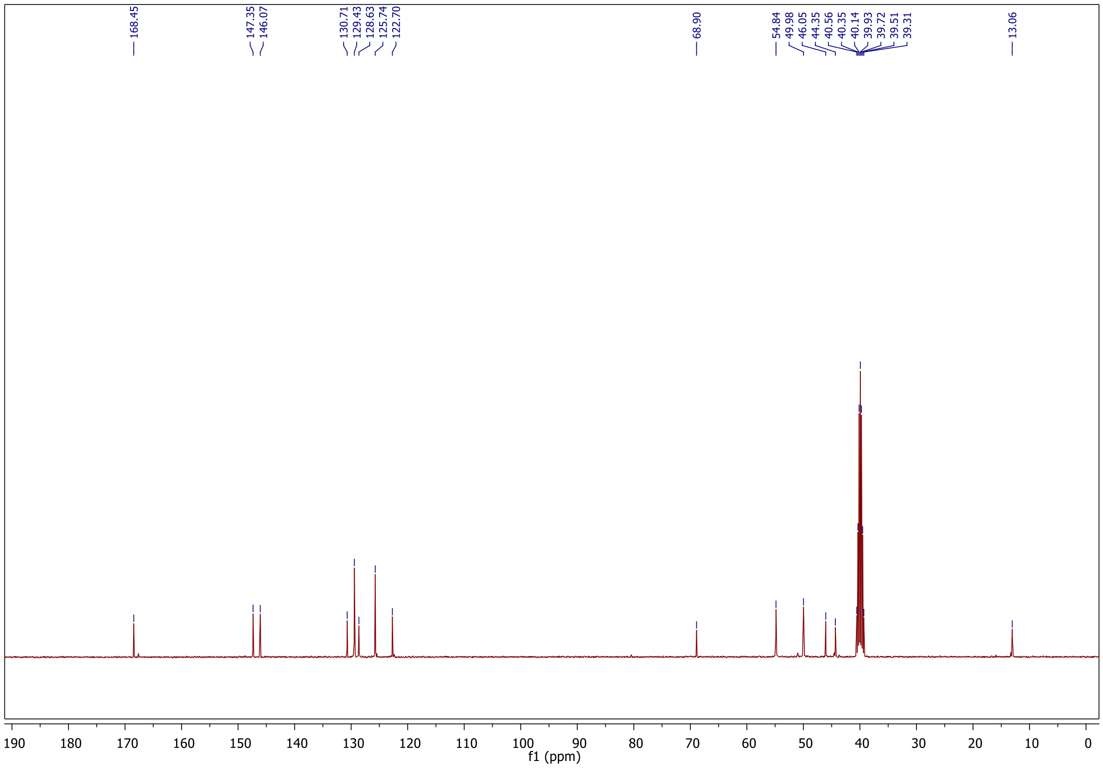

Figure S16. ^{13}C-NMR Spectrum of compound **14** (DMSO-*d*$_6$, 100 MHz).

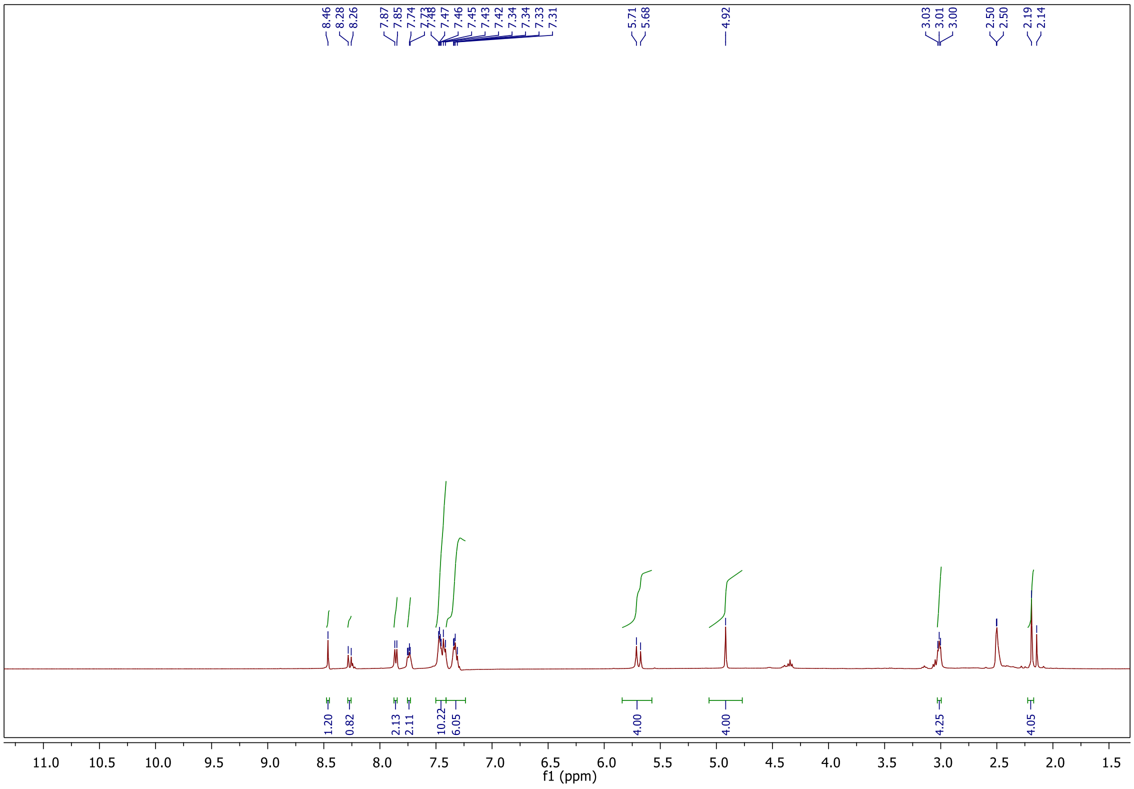

Figure S17. ^{1}H-NMR Spectrum of compound **15** (DMSO-d_6, 400 MHz).

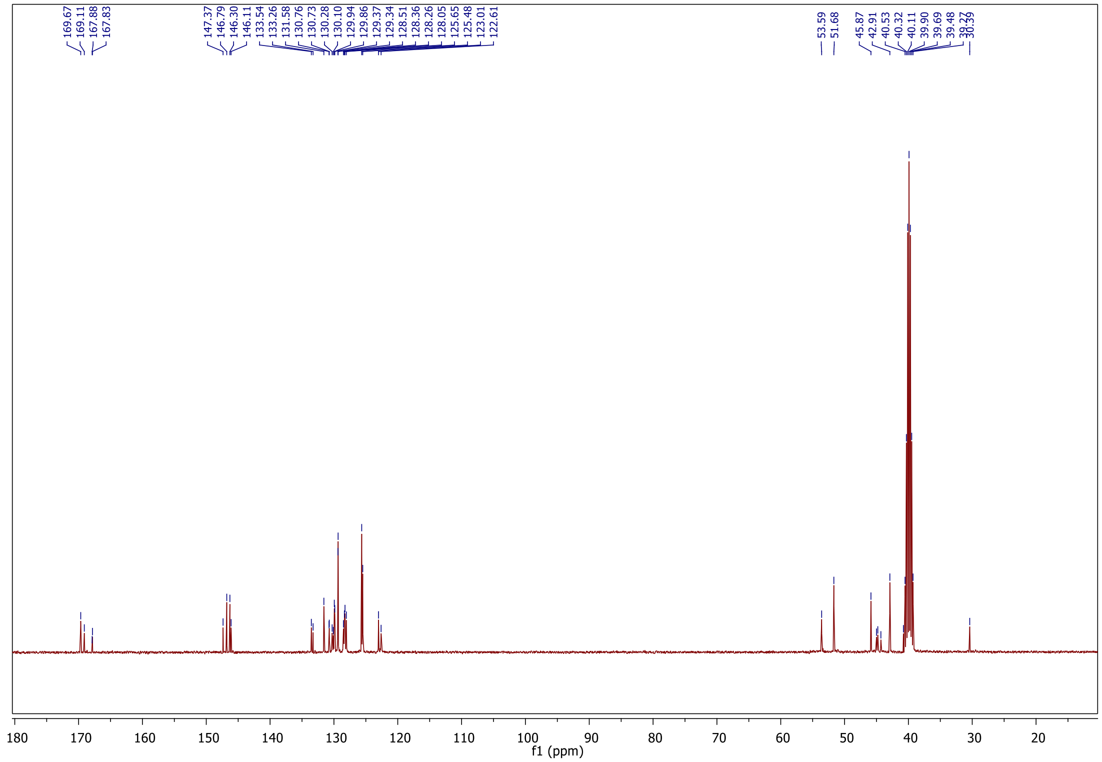

Figure S18. ^{13}C-NMR Spectrum of compound **15** (DMSO-d_6, 100 MHz).

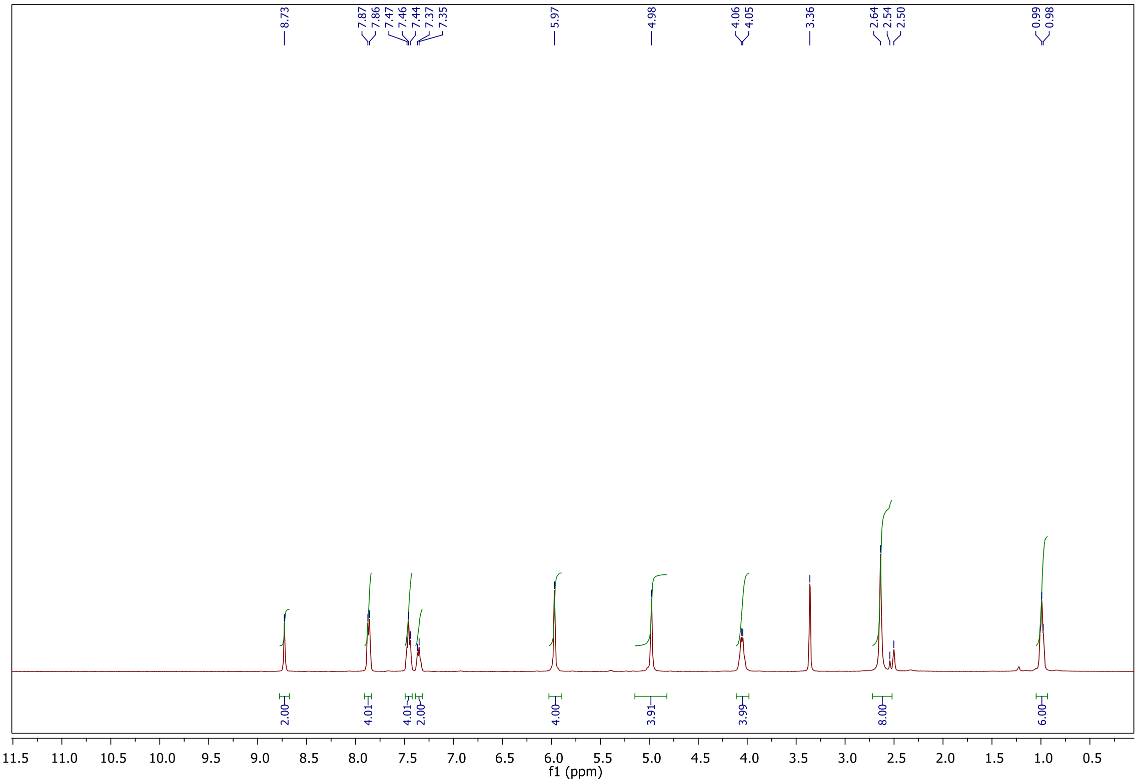

Figure S19. ^{1}H-NMR Spectrum of compound **16** (DMSO-d_6, 400 MHz).

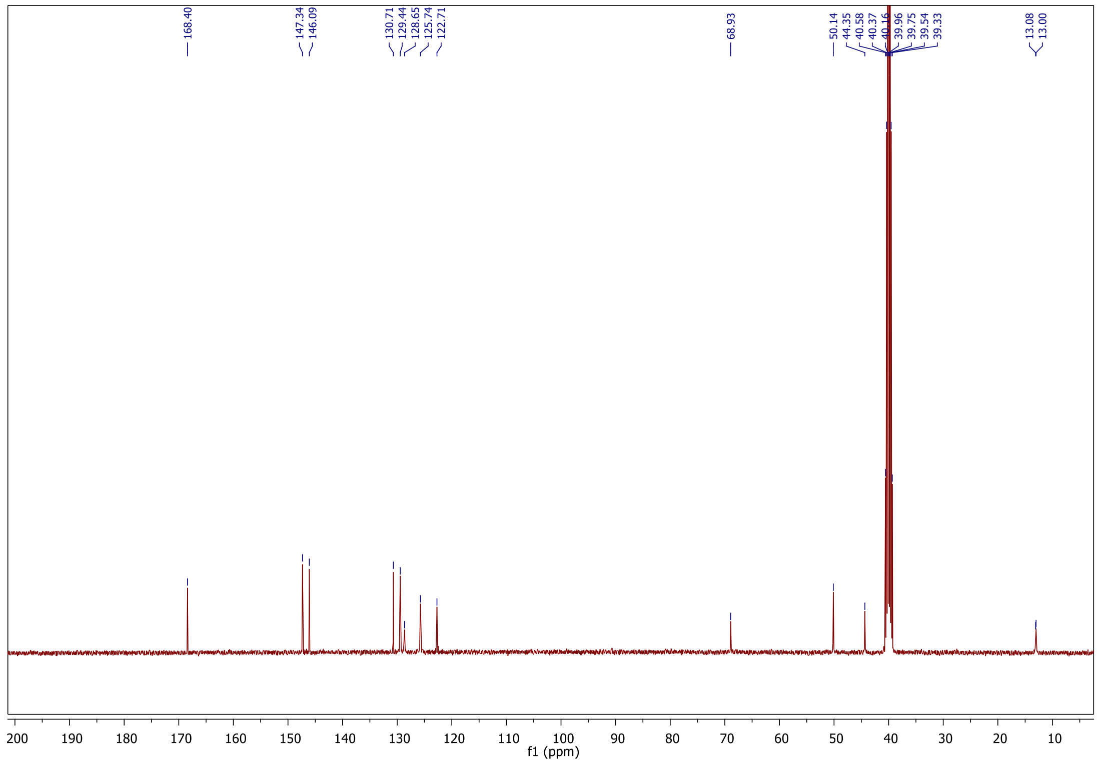

Figure S20. ^{13}C-NMR Spectrum of compound **16** (DMSO-d_6, 100 MHz).
